# Reanalysis of cancer mortality in Japanese A-bomb survivors exposed to low doses of radiation: bootstrap and simulation methods

**DOI:** 10.1186/1476-069X-8-56

**Published:** 2009-12-09

**Authors:** Greg Dropkin

**Affiliations:** 1 Flat 5, 32 Sheil Road, Liverpool L6 3AE, UK

## Abstract

**Background:**

The International Commission on Radiological Protection (ICRP) recommended annual occupational dose limit is 20 mSv. Cancer mortality in Japanese A-bomb survivors exposed to less than 20 mSv external radiation in 1945 was analysed previously, using a latency model with non-linear dose response. Questions were raised regarding statistical inference with this model.

**Methods:**

Cancers with over 100 deaths in the 0 - 20 mSv subcohort of the 1950-1990 Life Span Study are analysed with Poisson regression models incorporating latency, allowing linear and non-linear dose response. Bootstrap percentile and Bias-corrected accelerated (BCa) methods and simulation of the Likelihood Ratio Test lead to Confidence Intervals for Excess Relative Risk (ERR) and tests against the linear model.

**Results:**

The linear model shows significant large, positive values of ERR for liver and urinary cancers at latencies from 37 - 43 years. Dose response below 20 mSv is strongly non-linear at the optimal latencies for the stomach (11.89 years), liver (36.9), lung (13.6), leukaemia (23.66), and pancreas (11.86) and across broad latency ranges. Confidence Intervals for ERR are comparable using Bootstrap and Likelihood Ratio Test methods and BCa 95% Confidence Intervals are strictly positive across latency ranges for all 5 cancers. Similar risk estimates for 10 mSv (lagged dose) are obtained from the 0 - 20 mSv and 5 - 500 mSv data for the stomach, liver, lung and leukaemia. Dose response for the latter 3 cancers is significantly non-linear in the 5 - 500 mSv range.

**Conclusion:**

Liver and urinary cancer mortality risk is significantly raised using a latency model with linear dose response. A non-linear model is strongly superior for the stomach, liver, lung, pancreas and leukaemia. Bootstrap and Likelihood-based confidence intervals are broadly comparable and ERR is strictly positive by bootstrap methods for all 5 cancers. Except for the pancreas, similar estimates of latency and risk from 10 mSv are obtained from the 0 - 20 mSv and 5 - 500 mSv subcohorts. Large and significant cancer risks for Japanese survivors exposed to less than 20 mSv external radiation from the atomic bombs in 1945 cast doubt on the ICRP recommended annual occupational dose limit.

## Background

Analyses of cancer mortality in survivors of the atomic bombing of Japan in 1945 have played a central role in establishing risk estimates and radiation protection limits for workers and the wider public. While many people exposed at Hiroshima and Nagasaki received fairly high doses, the recommended occupational and environmental dose limits aim to control the risks from much lower doses. Typically, analyses of the full cohort of Japanese survivors, whose doses ranged from 0 - 4000 mSv or more, are extrapolated downwards to predict the risks from doses below 20 mSv [1]. However, even if a particular model gives a good overall account of the full range of data, fitting that model and extrapolating the results may misrepresent the risks at low doses where the dose response may be quite distinct.

My previous paper [2] considered the cancer mortality data for those survivors whose external (flash) dose from the A-bombs was below 20 mSv, the annual occupational dose limit recommended by the International Commission on Radiological Protection (ICRP). I applied several different models, each of which used as their exposure variable the radiation dose lagged by an unknown latency. One model adopted the standard assumption that Excess Relative Risk (ERR) is proportional to dose, in this case the lagged dose. Other models allowed that ERR might be a non-linear function of dose, as suggested by radiation cell biology studies of the bystander effect. All the models detected ERR values for a dose of 10 mSv which were far higher than predicted by extrapolating the standard analyses of the full cohort. The linear model found large, significant results for the liver, while the preferred non-linear model found large, significant results for the stomach, liver, and lung. These results occurred over a range of latencies.

The statistical method I used to assess the significance of the findings with non-linear models was criticised by Mark Little [3]. In this paper the data is re-analysed with other methods of statistical inference for the preferred non-linear model.

The models use Poisson regression to analyse the grouped data in the 0 - 20 mSv subcohort of the 1950-1990 Life Span Study LSS12 [4]. The models take the form λ = λ_o_(1+ERR) where λ is the cancer risk, λ_o _depends only on control variables, and ERR depends only on the lagged radiation dose. Profile Likelihood Confidence Intervals for ERR were based in [2] on the Likelihood Ratio Test (LRT) for comparisons between nested models. Once latency is fixed, the models depend smoothly on all other parameters. If Wilks Theorem [5] holds, the asymptotic null distribution of LRT is χ^2 ^on d degrees of freedom when the null hypothesis is specified by fixing the values of d parameters in the wider model. For example, the LRT comparison of the linear model ERR = βD with the control model ERR = 0 is asymptotically χ^2 ^on 1 d.f. if the control model holds. Here the null hypothesis is β = 0 and Wilks Theorem does apply to this nested pair. As Little [3] pointed out, the non-linear models are less well behaved. The transient model ERR = σDexp(-τD) and the two-phase model ERR = βD + σDexp(-τD) are indeterminate in τ when σ = 0, so one of the regularity conditions for Wilks Theorem fails. In any case, even if the asymptotic null distribution of LRT were known, the distribution for a given finite set of records may differ from the asymptotic case.

However, the actual distribution of LRT can be estimated by simulation before comparing two competing models or constructing confidence intervals. Bootstrap methods [6,7] can also be used to construct confidence intervals more directly.

In response to questions raised during peer review I also consider whether the data justifies fitting the two-phase model, investigate uncertainties in the "optimal latency" as estimated from the data, extend the analysis to include the covariate "city", and compare results from the 0 - 20 mSv and 5 - 500 mSv dose ranges.

## Methods

An initial analysis of latency is carried out for all cancer sites with at least 100 cases (deaths) in the 0 - 20 mSv subcohort. Sites which appear to have a raised dose response are then analysed by the bootstrap and the distribution of LRT is estimated by simulation. A fuller simulation is carried out for the stomach, the site with the largest number of cases in this subcohort. The analysis focuses on the behaviour of the two-phase model (defined below) and its relation to the linear model. It aims to compare confidence intervals obtained by bootstrap and LRT simulation methods and to discover without relying on Wilks Theorem, whether the apparent elevation and non-linearity of the dose response in the 0 - 20 mSv subcohort are statistically significant. Further analysis considers variation in latency, the impact of "city", male and female subcohorts, and the 5 - 500 mSv dose range. Computations use the statistical freeware **R **[8], giving a further check on previous results. Possible sources of error in the Japanese data itself and the potential for confounding by other covariates as discussed in [2], are not reconsidered here.

As previously source data for the 1950-1990 mortality cohort LSS12 was obtained from RERF [9] via the CEDR [10], and the 0 - 20 mSv subcohort defined by restricting the weighted adjusted colon dose. The subcohort comprises 3011 data cells with 1690391.75 p-y. The control model, linear model, and two-phase model are defined as in [2]. Briefly, the control model is log-linear in 14 indicator variables for 5 year age-at-exposure categories, an indicator for gender, and the numerical variable log mean attained age, each of which is well-defined for the data cells. The control model is specified as λ = λ_0 _= exp(α+Σβ_j_x_j_) where α and the β_j _are unknown parameters, x_j _are the covariates, exp denotes exponential, and the Poisson parameter in cell i with T_i _person-years is λ_i_T_i _where λ_i _is evaluated using the covariates in cell i. At this stage, as in [2], "city" (Hiroshima or Nagasaki) is not included as a covariate.

Other models are defined by λ = λ_0_(1+ERR) where ERR depends only on D_ϕ_, the radiation dose lagged by a latency parameter ϕ. D_ϕ _is defined as the weighted adjusted colon dose in 10 mSv units when time-since-exposure ≥ ϕ, and 0 otherwise.

For the linear model, ERR = βD_ϕ_

For the two-phase model, ERR = βD_ϕ _+ σD_ϕ_exp(-τD_ϕ_) where exp denotes exponential.

The models require 1+ERR > 0 in all cells so that the Poisson distributions are defined. For the two-phase model τ > 0, slightly modifying the previous approach where τ ≥ 0. However, τ = 0 reduces to the linear model as does σ = 0.

In fitting a model at latency ϕ by Poisson regression, the unknown parameters α,β_j _and β, σ, τ (as appropriate to the model) are chosen to maximise the likelihood of the observed data assuming independent Poisson distributions in each cell. Equivalently, fitting minimises the sum over the 3011 data cells of E_i _- O_i_ln(E_i_), where O_i _and E_i _= λ_i_T_i _are the observed and expected number of cases in cell i.

Latency ϕ is fixed during the minimisation. The models depend smoothly on all other parameters. ERR_D,ϕ _denotes the Excess Relative Risk evaluated at D for the fitted model with latency ϕ. Note ERR_1,ϕ _is the estimated risk at 10 mSv lagged by ϕ years. For model I nested within model J, LRT_J-I,ϕ _is the Likelihood Ratio Test computed at latency ϕ.

Code written in **R **emulates the previous model fitting with Excel-Solver, using the Newton-Raphson minimisation routine "optim" [11]. Results for the stomach at latency ϕ = 5, 6,... 44 are checked against the values obtained previously.

At a given cancer site, the control, linear, and two-phase models are fitted at latency ϕ = 5, 6,... 44 to give LRT_J-I,ϕ _for each pair of nested models: linear against control, two-phase against control, and two-phase against linear. Sites are selected for further analysis if LRT_lin-con,ϕ _> 4 for some ϕ, or if LRT_2p-lin,ϕ _> 6 for some ϕ (not assuming that this criterion establishes statistical significance). For sites meeting the criterion for the linear model, Profile Likelihood Confidence Intervals for β = ERR_1,ϕ _are computed using Wilks Theorem at integer values of ϕ. For sites meeting the criterion for the two-phase model, ϕ = ϕ_mabs _is chosen to maximise LRT_2p-con,ϕ _(not LRT_2p-lin,ϕ_) so that fitting at ϕ_mabs _gives the absolute Maximum Likelihood Estimate (MLE) for the model. The "optimal latency" ϕ_m _is chosen to maximise LRT_2p-con,ϕ _subject to the constraint ERR_1,ϕ _≥ 0. Bootstrap confidence intervals and LRT simulations are then computed at ϕ_m _. Details are given in the next two more technical subsections, and examples with commentary are supplied with Additional File 1. The method for taking account of uncertainty in the "optimal latency" is outlined in a final subsection.

### Bootstrap

Fitting the two-phase model at ϕ = ϕ_m _gives a maximum likelihood estimate  for the vector of all 20 model parameters and determines fitted values of λ_i _in each data cell. The parametric bootstrap assumes the observed data arose from sampling this fitted model, and considers the variation arising if other data had been sampled from that same model.

Sampling the independent Poisson distributions with parameters λ_i_T_i _gives simulated observations in each data cell. Fitting the two-phase model to this simulated data gives simulated parameter values including ,  and , and therefore a simulated value of ERR = ERR_D,ϕ _= ). The entire process is repeated B times to give the bootstrap replications ERR*.

There are many well-established methods to obtain confidence intervals from bootstrap replications [7,12]. The two options chosen here are the original "percentile" method, which is easy to describe, and the "Bias-corrected accelerated" method which is much faster to compute, and in general more accurate.

The 1-2α "percentile" confidence interval has endpoints

where  denotes the Bα^th ^ordered value of ERR*. With B = 1000 replications and α = 0.025, the endpoints of the 0.95 percentile interval are the 25^th ^and 975^th ^order statistics of ERR*.

For the Bias-corrected accelerated (BC_a_) confidence interval (see [7] Chapter 5 pp. 203-207 and p.249) simulation is again carried out using λ_i_T_i_. However, the simulated observations are now used to fit the model to the "Least Favourable family" of distributions, obtained by restricting the parameter vector ψ to a line parametrised by ζ, passing through the MLE  and defined by  where

Here i^-1^() is the inverse of the Fisher Information evaluated at  and  is the gradient of the function h which defines the variable of interest (in this case ERR) in terms of the model parameters ψ.

For each bootstrap replication, fitting the simulated data along the LF family determines ζ and thus the simulated value ERR = . If ERR*_LF _denotes the resulting set of B replications, the BC_a _confidence interval has endpoints

where

Here Φ is the standard normal cumulative distribution and z^(α) ^is the 100αth percentile point of a standard normal distribution. The parameters *w *and *a *are estimated from the bootstrap replicates. The bias- correction *w *is computed as

i.e. the fraction of bootstrap replications along the LF family which are below the original fitted value of ERR, in normal units.

The acceleration *a *is computed as

where m_3 _and m_2 _are the 3^rd ^and 2^nd ^moments of , the partial derivative of the log likelihood of the simulated data with respect to ζ, evaluated at ζ = 0.

Code for this calculation is shown in Additional File 1, Code File **AC1.txt**, which calls Additional File 1, Data Files **stomdat1.txt**, **lambda2.txt **and **theta2.txt**. Additional File 1, Commentary File **ACom1.doc **discusses this code.

Despite the intricate formulas, computation is much faster for BC_a _than for the simpler percentile method, because optimisation is now restricted to a line within the full 20-dimensional parameter space. The percentile CIs are computed at B = 1000, the recommended minimum number, while BC_a _results are obtained at B = 5000.

Coverage for the percentile and BC_a _methods is tested using the stomach data. For each of 400 sets of simulated observations generated by sampling the Poisson distributions determined by the original fitted parameters, the model is refitted to the simulated data. Confidence intervals for the simulated ERR are obtained by the bootstrap methods (B = 1000) using the refitted parameters and counted as 1 or 0 if they contain or exclude the "true" value of ERR from the original fitted parameters. To streamline these computations of coverage for the percentile method, data is restricted to the Hiroshima subcohort (N = 1536 cells) and the model simplified to use 10-year rather than 5-year age-at-exposure categories. Estimating coverage for the BC_a _method uses the full 0 - 20 mSv dataset and model. The application of both methods to compute CIs from the observed data uses the full dataset and model.

### Simulation of LRT

The Likelihood Ratio Test (LRT) for comparing nested models H_0 _≤ H_1 _(H_0 _contained within H_1_) given the observed data  is LRT = 2[ℒ(H_1_, ) - ℒ(H_0_, )] where ℒ(H_i_, ) is the log likelihood of the observed data after fitting the model H_i_. In well-behaved situations where Wilks theorem [5] applies, the asymptotic distribution of LRT is known, provided the data was drawn from a distribution which satisfies the null hypothesis H_0_. In particular, an appropriate critical value t_0.95 _can be chosen for rejecting H_0 _if LRT > t_0.95 _and this will result in a 5% error rate of rejecting H_0 _when it is true. Typically H_0 _fixes the values of d parameters in the full model H_1 _which allows those parameters to vary freely in an open neighbourhood. In this case Wilks theorem says that LRT is asymptotically χ^2 ^distributed on d degrees of freedom if the observed data was generated by the model with those d parameters fixed as specified. On that basis, the null hypothesis can be rejected if LRT > t_0.95 _= F^-1^(0.95) where F is the cumulative of a χ^2 ^distribution on d degrees of freedom.

Wilks theorem depends on many regularity conditions for the models H_0 _and H_1_. If these fail, LRT may not be χ^2 ^distributed; its distribution may depend on H_0 _rather than just on the number of specified parameters, and there may be no simple universal 95% critical value for rejecting H_0_. Simulation aims to estimate the distribution of LRT and then to obtain conservative critical values which are sufficient to reject H_0_. Confidence intervals for functions of the parameters are constructed using these critical values.

For the two-phase model  is the Likelihood Ratio Test for the hypothesis H_0_: (β, σ, τ) = (β_0_,σ_0_,τ_0_) vs. the alternative (β, σ, τ) ≠ (β_0_,σ_0_,τ_0_), i.e. H_1 _is the union of H_0 _with its alternative, and leaves (β, σ, τ) free to vary, subject only to the defining conditions of the two-phase model. For various choices of H_0 _the null distribution of  assuming H_0 _holds is sampled by Monte Carlo simulation (see [7] Chapter 4; for another context see [13] p.84) as follows.

One) The model is fitted subject to H_0_. The resulting parameter estimates give fitted values  and therefore  T_i _for each cell, subject to H_0_.

Two) Poisson distributions with parameters  T_i _are sampled independently to give simulated cases  in each cell

Three) The model is fitted to the simulated data  subject to H_0_, yielding new fitted values  for each cell. Put .

Four) The model is fitted to the simulated data  with (β, σ, τ) allowed to vary freely within the parameter space, yielding new fitted values  for each cell. Put K = . The simulated value of  is defined as LRT* = K_0 _- K. Note that up to a constant which depends on the simulated data  but not on the model, K_0 _= -2ℒ(H_0_, ) while K = -2ℒ(H_1_, ).

Five) Steps Two through Four are repeated 500 (or 1000) times to give the sample LRT*

An example of the required code is given in Additional File 1, Code File **AC2.txt **which calls Additional File 1, Data Files **stomdat1.txt**, **lambda1.txt**, **theta1.txt**, and Additional File 1, Code File **AC2s.txt. **Additional File 1, Commentary File **ACom2.doc **discusses this code.

While step Four is time-consuming, steps One and Three are quick as the model is log-linear in the remaining parameters. Step Two is trivial in **R**. Note that in step Four the possible values of (β, σ, τ) are still constrained by the requirement that 1+ERR > 0 in all cells. For computability, the constraint is set at 1+ERR ≥ 0.001.

To check whether Wilks theorem holds, LRT* is first tested against a χ^2 ^distribution on 3 d.f. by the Kolmogorov-Smirnov (one-sample) test. LRT* is then tested against gamma distributions with unknown shape and rate parameters. A γ distribution is fitted to LRT* using the "fitdistr" subroutine of **R **(MASS library) [14]. When testing LRT* against the *fitted *distribution with shape parameter  and rate parameter , the Kolmogorov statistic D is obtained from the (one sample) K-S test but its p-value is determined by simulation (as in the Lilliefors test). When LRT* has 500 elements, 500 random deviates are taken from the fitted γ distribution with parameters  and  to form a sample S, a new gamma distribution Γ is fitted to S, the K-S statistic D* for testing S against Γ (one-sample) is computed, and the process is repeated 5000× to estimate the probability that D* > D when samples are drawn from γ(, ). **R **code was adapted from [15].

For the special case (β_0_,σ_0_,τ_0_) = (0,0, arbitrary) the sample LRT* uses 1000 replications; note that in this case  = LRT_2p-con_. Likewise at the MLE estimate (β_0_,σ_0_,τ_0_) = (), LRT* is produced during the (percentile) bootstrap with 1000 replications. In both cases the simulations required for the p-value of D use 1000 random deviates of the fitted γ distribution.

For the liver, lung, pancreas and leukaemia, LRT simulation (×1000) is carried out at (0,0, arbitrary) and (), and (×500) at 3 points chosen at random with σ_0 _= 0 and β_0 _in the intervals -0.5 < β_0 _< 0, 0 < β_0 _< 1, and 1 < β_0 _< 10, and also at 4 points chosen at random in a neighbourhood N_10 _of (). N_10 _comprises those (β_0_,σ_0_,τ_0_) for which the observed (not simulated)  < 10. Each set of simulations gives a sample **LRT**_i_* and corresponding fitted gamma distributions γ_i_

For the stomach, simulations and corresponding fitted γ_i _are carried out at the MLE and 50 other random points in the parameter space: (×500) at 11 points with σ_0 _= 0, 4 points in a neighbourhood N_10 _of (), 34 other points in the (β, σ, τ) parameter space, and (×1000) at (0,0, arbitrary) and ().

Estimates of a 95% critical value for the null distribution of LRT are then obtained in three ways. For each cancer site, the fitted gamma distributions and the order statistics of the simulated **LRT**_i_* are used to estimate a global 95% critical value as  = max(sup(t_.95,i_), sup(s_.95,i_)) where t_.95,i _is the 95^th ^percentile of γ_i _and s_.95,i _is the 95^th ^percentile of **LRT**_i_*. That is, at each value of (β_0_,σ_0_,τ_0_) tested for a particular site, 95% of the simulated LRT values and the 95^th ^percentile of the fitted gamma distribution are both below . An overall conservative estimate t_.95 _is chosen to exceed the corresponding maximum for all simulations and all cancer sites investigated. At each site, a refined estimate  = max(sup(t_.95,j_)) is obtained by considering only the fitted gamma distributions and restricting to simulations at points (β_0_,σ_0_,τ_0_) within N_10_. At each cancer site  ≤  ≤ t_.95_.

The neighbourhood N_10 _of () is termed "appropriate" if the overall estimate t_.95 _≤ 10 so that for (β_0_,σ_0_,τ_0_) outside N_10_,  > t_.95 _. This is not a circular definition; t_.95 _is computed from the behaviour of simulated LRT at points outside as well as inside N_10 _for each site, while for (β_0_,σ_0_,τ_0_) within N_10 _and even at () simulation may, if N_10 _is inappropriate, give LRT* with 95^th ^percentile > 10.

Profile Likelihood Confidence Intervals for ERR_1,ϕ _and ERR_0.025,ϕ _are then constructed using all three estimates for the critical value. With ϕ fixed, let U_ϕ _be the set of those (β_0_,σ_0_,τ_0_) which cannot be ruled out with 95% confidence as  < t_.95_(alternatively <, or <) Define the 95%CI for ERR_D,ϕ _as the range of ERR_D,ϕ _over U_ϕ_.

When σ_0 _= 0,  is the Likelihood Ratio Test for the hypothesis H_0_: β = β_0 _and σ = 0 vs. the alternative β ≠ β_0 _and σ = 0, while LRT_2p-lin _is the Likelihood Ratio Test for the hypothesis σ = 0 vs. the alternative σ ≠ 0. Note LRT_2p-lin _+  = . Simulated null distributions of all three LRT's are obtained for points with σ = 0. Conservative, global, and refined estimates T_.95_,  and  of the 95% critical value for LRT_2p-lin _are then defined in the same fashion as t_.95_,  and  for . For each cancer,  is the supremum of the 95^th ^percentiles of γ distributions fitted to the simulations of LRT_2p-lin_,  is the supremum of 95^th ^percentiles of the fitted γ distributions and simulations of LRT_2p-lin_, and T_.95 _is an overall conservative value exceeding  for all cancer sites considered. Likewise  and  are defined from the 99^th ^and 99.9^th ^percentiles of the fitted γ distributions. These various estimates are used as critical values for LRT_2p-lin _when testing the linear model against the two-phase model, given the observed data.

### Latency

For latency ϕ = 5, 6,... 44 BC_a _95%CIs for ERR_1,ϕ _and ERR_0.025,ϕ _are computed.

Since the observed data is regarded as a sample from an underlying distribution, the optimal latency ϕ_m _as inferred from the data is an estimate, whose distribution is again obtained by simulation. The two-phase model is fitted at ϕ_m _and the fitted model is resampled to provide simulated data, from which a new estimate  is determined. To simplify,  is restricted to integer values 5, 6,... 44. The process is repeated ×200 for the stomach, liver, lung, pancreas and leukaemia. The resulting set of  is compared with the range of latencies for which BC_a _95%CIs are strictly positive, and likewise with the range for which LRT_2p-lin _exceeds the estimated critical values.

To test whether the distributions of LRT_2p-con _and LRT_2p-lin _depend on latency, null distributions at (β_0_,σ_0_,τ_0_) = (0,0, arbitrary) are simulated (×500) for the stomach with ϕ = 5, 10, 15,... 40 and compared with the null distribution from simulation (×1000) at ϕ_m_.

### City, dose range, and gender

The models are refitted with "city" as an added control covariate. For each of the 5 cancers, the optimal latency Φ_m _(restricted to 5, 6,... 44) is determined along with latency ranges over which BC_a _95% and 90% CIs for ERR_1 _are strictly positive.

The models including "city" are fitted to the 5 - 500 mSv subcohort. A limited analysis of the null distributions of LRT_2p-con _and LRT_2p-lin _including "city" is carried out on the 0 - 20 mSv and 5 - 500 mSv dose ranges. For each cancer at its Φ_m_, H_0_: (β_0_,σ_0_,τ_0_) = (0,0, arbitrary) is used for simulation of LRT_2p-con _and H_0_: (β_0_,σ_0_,τ_0_) = (,0, arbitrary) where  is the fitted parameter for the linear model, is used for simulation of LRT_2p-lin_. Gamma distributions fitted to the simulated LRT* are used to estimate 95% critical values and to assign p-values to the LRT_2p-con _and LRT_2p-lin _arising from the observed data.

The models including "city" are fitted separately to the male and female subcohorts in the 0 - 20 mSv dose range for the stomach, liver, and lung.

Finally, the linear model including "city" is fitted to the 0 - 5 mSv, 5 - 20 mSv, 0 - 0.5 mSv, and 0.5 - 20 mSv data for the liver and lung.

The very extensive simulations of LRT were shared across a number of PC's.

## Results

Table 1 shows the individual cancer sites with over 100 cases (deaths) in the subcohort.

**Table 1:** Cancer sites with over 100 cases (deaths) in the 0-20 mSv subcohort

Site	Cases (deaths) in the 0-20 mSv subcohort
Stomach	1482
Liver	540
Lung	524
Pancreas	179
Rectum	171
Oesophagus	131
Gall	125
Lymph	109
Leukaemia	105
Breast (F)	105
Urinary	103

To check **R **against Excel, the two-phase model is fitted to the stomach data for latencies ϕ = 5, 6,... 44 and LRT_2p-con,ϕ _computed in both programmes. The difference (LRT_2p-con,ϕ,R_) - (LRT_2p-con, ϕ, Excel_) has a range of (-0.079, 0.000), mean = -0.004, s.d. = 0.014. At the optimal latency ϕ_m _= ϕ_mabs _= 11.89, LRT_2p-con, ϕ _= 21.1903 by either method. The parameter estimates in **R **are  = 0.4586,  = 120.0076,  = 82.6729, confirming the point estimates reported in [2]. On that basis, the methods are taken as comparable. Results are also comparable when computing LRT-based confidence intervals.

**Figure 1:**
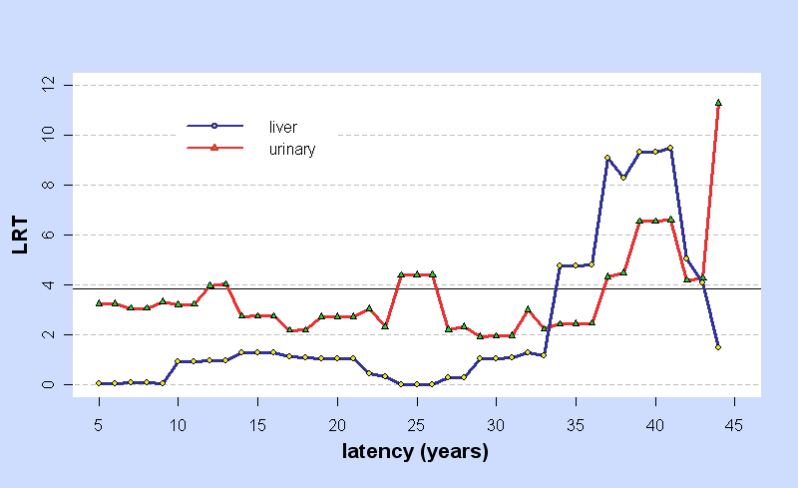
**LRT for Linear vs. Control model: liver and urinary**. For cancers of the liver (blue) and urinary (red) and for latency ϕ = 5, 6,... 44 years, LRT = LRT_lin-con,ϕ _is the Likelihood Ratio Test for comparing the linear and control models at latency ϕ. Values above the horizontal line LRT = 3.841 are significant at p < 0.05.

**Figure 2:**
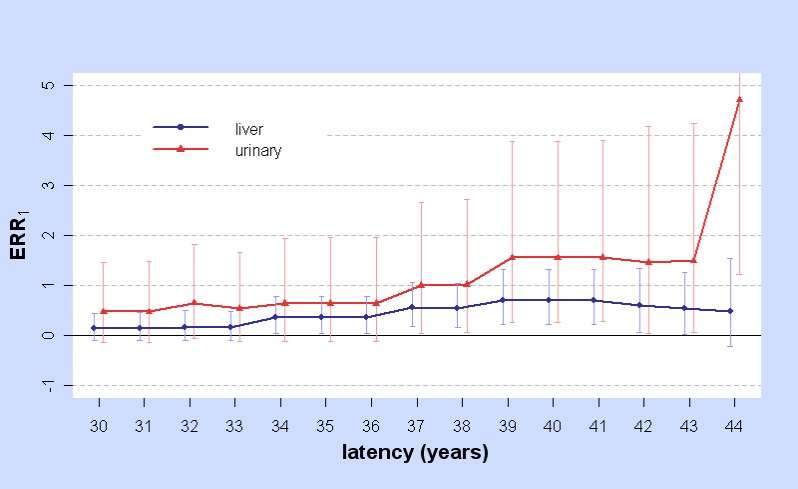
**ERR_1 _with 95%CIs for Linear model: liver and urinary**. For cancers of the liver (blue) and urinary (red) and for latency ϕ = 30, 31,... 44 years, ERR_1 _is the Excess Relative Risk from 10 mSv (lagged dose) as estimated by fitting the linear model at latency ϕ. The error bars show Profile Likelihood 95%CIs assuming a χ^2 ^(1 d.f.) distribution of LRT for testing β = β_0 _against β ≠ β_0_.

For the linear model ERR = βD_ϕ _with ϕ = 5, 6,... 44 only the liver and urinary tract showed significant results. Figure 1 shows LRT_lin-con,ϕ _against ϕ for these two cancers, while Figure 2 shows the fitted values ERR_1 _=  when ϕ ≥ 30 with 95%CIs (Profile Likelihood, assuming LRT ~ χ^2 ^on 1 d.f. for testing β = β_0 _against β ≠ β_0_). For the liver with this model, ϕ_m _= ϕ_mabs _= 38.58, at which  = 0.69 (0.25, 1.26). Of the 540 cases in the subcohort, 171 have ϕ > 38.58. For the urinary tract, ϕ is locally optimal at 41.32 at which  = 2.14 (0.51, 5.12). Of the 103 cases in the subcohort, 23 have ϕ > 41.32.

**Figure 3:**
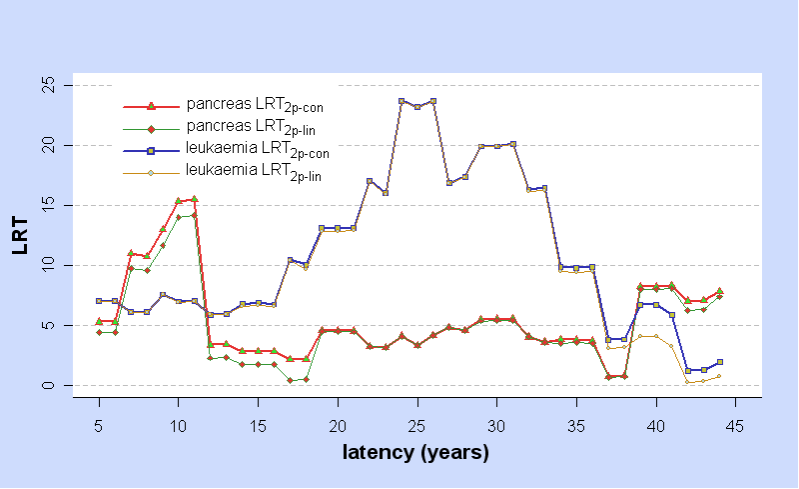
**LRT for Two-phase vs Control and Linear models: pancreas and leukaemia**. For each cancer and for latency ϕ = 5, 6,... 44 years, LRT_2p-con _= LRT_2p-con,ϕ _is the Likelihood Ratio Test for comparing the two-phase and control models at latency ϕ, and LRT_2p-lin _= LRT_2p-lin,ϕ _is the Likelihood Ratio Test for comparing the two-phase and linear models at latency ϕ.

**Figure 4:**
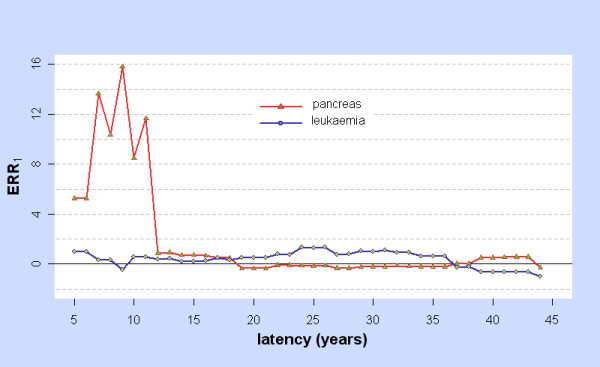
**ERR_1 _for Two-phase model: pancreas and leukaemia**. For cancers of the pancreas (red) and leukaemia (blue) and for latency ϕ = 5, 6,... 44 years, ERR_1 _is the Excess Relative Risk from 10 mSv (lagged dose) as estimated by fitting the two-phase model at latency ϕ.

The stomach, liver, lung, pancreas, and leukaemia were selected for further analysis as LRT_2p-lin,ϕ _> 6 for some ϕ, an indication that the dose response may be non-linear. For the pancreas and leukaemia, Figure 3 shows LRT_2p-lin,ϕ _and LRT_2p-con,ϕ _while Figure 4 shows ERR_1,ϕ _=  as estimated from fitting the two-phase model at latency ϕ. Note that  may differ greatly from the value obtained by fitting the linear model at ϕ. Related graphs for the stomach, liver, and lung were shown in [2], though their statistical significance is reconsidered below.

Table 2 shows the latencies ϕ_mabs _and ϕ_m _which maximise LRT_2p-con,ϕ _without and with the constraint ERR_1,ϕ _≥ 0, the values of LRT_2p-con,ϕ _and LRT_2p-lin,ϕ _at ϕ_m _along with the fitted parameters and corresponding point estimates of ERR_1 _and ERR_0.025 _and their bootstrap confidence intervals by the BC_a _(5000 replications) and percentile (1000 replications) methods. For the stomach, liver, and lung, these confirm the point estimates in [2].

**Table 2:** Latencies, LRT, fitted parameters, ERR and bootstrap confidence intervals for the two-phase model

Site	**ϕ**_**mabs**_	**ϕ**_**m**_						**ERR**_**1**_	**ERR**_**0.025**_
Stom	11.89	11.89	21.19	19.64	0.46	120.01	82.67	0.46 (0.21, 0.80)^bca^ (0.19, 0.79)^per^	0.39 (0.15, 0.70)^bca^ (0.15, 0.71)^per^

Liver	36.90	36.90	34.87	27.64	1.43	291.22	76.76	1.43 (0.70, 2.41)^bca^ (0.72, 2.39)^per^	1.10 (0.49, 1.90)^bca^ (0.40, 1.88)^per^

Lung	36.99	13.60	16.04	14.74	0.44	37.90	4.46	0.88 (0.34, 1.50)^bca^ (0.27, 2.07)^per^	0.86 (0.40, 1.49)^bca^ (0.32, 2.02)^per^

Panc	11.86	11.86	16.22	14.80	9.77	1060.94	43.77	9.77 (3.50^a^, 14.27)^bca^ (1.72, 55.11)^per^	9.12 (3.25^b^, 13.46)^bca^ (1.49, 48.25)^per^

Leuk	23.66	23.66	25.40	25.29	1.69	3353.15	172.25	1.69 (0.20, 4.81)^bca^ (0.19, 4.77)^per^	1.17 (0.18, 3.58)^bca^ (0.24, 3.79)^per^

ϕ_mabs_: latency (years) which maximises **LRT**_**2p-con**_

ϕ_m_: optimal latency, which maximises **LRT**_**2p-con **_with the constraint ERR_1 _≥ 0

: Likelihood Ratio Test for the two-phase model against the control model at ϕ_m_

: Likelihood Ratio Test for the two-phase model against the linear model at ϕ_m_

: fitted parameters for the two-phase model at ϕ_m_

ERR_1 _: Excess Relative Risk at 10 mSv (lagged by ϕ_m_), as estimated from the fitted two-phase model

ERR_0.025 _: Excess Relative Risk at 0.25 mSv (lagged by ϕ_m_), as estimated from the fitted two-phase model

^bca ^Bootstrap BC_a _95%CI (5000 bootstrap replications)

^per ^Bootstrap percentile 95%CI (1000 bootstrap replications)

^a ^: minimum bootstrap value, lower tail probability = 0.034

^b ^: minimum bootstrap value, lower tail probability = 0.024

Coverage for the BC_a _method is estimated for the stomach at 94% for ERR_1 _(376 of 400) and 93% for ERR_0.025 _(372 of 400). Coverage for the percentile method is estimated for the stomach using the 0 - 20 mSv Hiroshima subcohort with the simplified two-phase model (10 year age-at-exposure categories) giving 94.3% for ERR_1 _(377 of 400) and 93% for ERR_0.025 _(372 of 400). All CIs reported in Table 2 use the full model (5 year categories, both cities).

**Figure 5:**
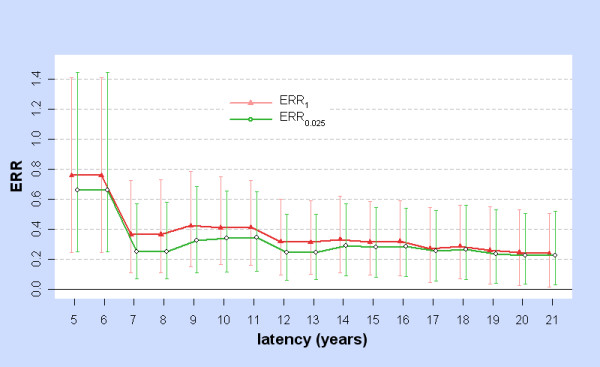
**ERR_1 _and ERR_0.025 _with 95%CIs for Two-phase model: stomach**. For stomach cancers and for latency ϕ = 5, 6,... 21 years, ERR_1 _(red) is the Excess Relative Risk from 10 mSv (lagged dose) as estimated by fitting the two-phase model at latency ϕ, while ERR_0.025 _(green) is the Excess Relative Risk from 0.25 mSv (lagged dose) as estimated by fitting the two-phase model at latency ϕ. The error bars show bootstrap 95%CIs computed with the BC_a _method, using 5000 replications.

For the stomach with latency ϕ = 5, 6,... 21, Figure 5 shows the BC_a _95% confidence intervals (B = 5000) for ERR_1 _and ERR_0.025_. Each of these CIs is strictly positive. Since the minimum time-since-exposure in the subcohort is 6.08 years, for ϕ = 5 or 6 the model involves no correction for latency and ERR is a function of the unmodified colon dose.

More generally, for each site (stomach, liver, lung, pancreas, leukaemia) there is a neighbourhood surrounding ϕ_m _over which BC_a _95% CIs for ERR_1 _and ERR_0.025 _are strictly positive, which was evaluated restricting to integer latencies and using B = 5000 bootstrap replications. For the stomach, both CIs are strictly positive when 5 ≤ ϕ ≤ 21. For the liver, both CIs are strictly positive when 32 ≤ ϕ ≤ 43. For the lung, both CIs are strictly positive when 7 ≤ ϕ ≤ 21. For the pancreas, both CIs are strictly positive when 5 ≤ ϕ ≤ 11. For leukaemia, the 95% CI for ERR_1 _is strictly positive when 24 ≤ ϕ ≤ 26, but the 95% CI for ERR_0.025 _is strictly positive when 19 ≤ ϕ ≤ 36.

For each site, simulating data (200 sets) from the fitted model at ϕ_m _and re-determining the optimal latency  (restricting to integer values) from the simulated data shows the uncertainty in ϕ_m _itself. For the stomach 5 ≤  ≤ 21 for 196 out of 200 simulated datasets, while 5 ≤  ≤ 21 for 187 out of 200 simulated datasets. For the liver, 32 ≤  ≤ 43 for 200 out of 200 simulated datasets, while 32 ≤  ≤ 43 for 197 out of 200 simulated datasets. For the lung, 7 ≤  ≤ 21 for 184 of 200, while 7 ≤  ≤ 21 for 173 of 200 simulated datasets (nb for the lung ϕ_m _≠ ϕ_mabs_). For the pancreas the results are weaker, as 5 ≤  ≤ 11 for 157 of 200, and 5 ≤  ≤ 11 for 151 of 200 simulated datasets. For leukaemia 24 ≤  ≤ 26 for 132 of 200 and 24 ≤  ≤ 26 for 130 of 200, but 19 ≤  ≤ 36 for 188 of 200 and 19 ≤  ≤ 36 for 184 of 200 simulated datasets.

Additional File 1, Tables **S1, S2**, and **S3 **give the simulated null distributions of LRT for the two-phase model with the null hypothesis H_0_: (β, σ, τ) = (β_0_,σ_0_,τ_0_) vs the alternative (β, σ, τ) ≠ (β_0_,σ_0_,τ_0_), for the stomach, liver, lung, pancreas, and leukaemia. Additional File 1, Table **S1 **shows simulation for each cancer at the fitted values (β_0_,σ_0_,τ_0_) = () and for randomly selected points in the neighbourhood N_10 _of the fitted values (see Methods). Additional File 1, Table **S2 **shows simulation for each cancer with σ_0 _= 0, where τ_0 _is arbitrary. Additional File 1, Table **S3 **shows additional simulation for the stomach when σ_0 _≠ 0, but (β_0_,σ_0_,τ_0_) is outside the neighbourhood N_10_. For the simulations in **S2**, Additional File 1, Table **S4 **shows the results for the linear model and the comparison with the two-phase model. These simulations are carried out at the optimal latency for the specific cancer.

As the control parameters are optimised subject to H_0 _before simulating LRT, perhaps other choices of control parameters while retaining H_0 _might yield different estimates of the null distribution of LRT subject to H_0_. However, four sets of simulations (×500) for the stomach with (β_0_,σ_0_,τ_0_) = (0, 0, arbitrary) using very different choices of control parameters and total cases expected, yielded fairly similar LRT* and fitted gamma distributions as shown in Table 3. Optimised control parameters for the observed data are shown in the last row. This is evidence that the null distribution of  is approximately independent of the parameters used to simulate the data, provided that H_0 _holds. In the terminology of [7] (Chapter 4 p. 139)  is an "approximate pivot" even though Wilks Theorem fails. In turn, this gives the basis for rejecting H_0 _given the observed data.

**Table 3:** Null distribution of LRT_2p-con _for the stomach with various control parameters

α	**β**_**1**_	**β**_**2**_	**β**_**3**_	**β**_**4**_	**β**_**5**_	**β**_**6**_	**β**_7_	**β**_**8**_	**β**_**9**_	**β**_**10**_	**β**_**11**_	**β**_**12**_	**β**_**13**_	**β**_**14**_	**β**_**15**_	**β**_**16**_	N	qlrt	s	r	qgam	expect
-5	0	0	0	0	0	0	0	0	0	0	0	0	0	0	0	0	500	8.23	2.38	0.64	8.32	11389.77
-15	1	2	0	0	0	0	0	0	0	0	0	0	0	0	0	0	500	8.61	2.36	0.63	8.50	7528.62
-8	-1	0	2	0	0	0	0	5	0	0	0	0	0	0	0	0	500	8.39	2.35	0.63	8.42	1418.11
-14	1	1	1	1	1	1	1	1	1	1	1	1	1	1	1	1	500	8.45	2.24	0.60	8.59	1013.12
-18.13	-0.84	3.11	-1.05	-0.77	-1.09	-0.69	-0.27	-0.31	-0.29	0.20	0.15	0.29	0.40	0.37	0.45	0.16	1000	8.88	2.42	0.60	8.96	1482.00

α,β_j _: specified values of control parameters

N: number of simulations

qlrt: 95^th ^percentile of

s, r: shape and rate parameters of the fitted gamma distribution for

qgam: 95^th ^percentile of the fitted gamma distribution for

expect: expected number of cases

The simulated null distributions of  from Additional File 1, Tables **S1, S2**, and **S3 **are then applied to estimate t_.95_,  and  as defined in Methods. All simulations showed LRT and its fitted gamma distribution had 95^th ^percentiles qlrt(0.95) < 10 and qgamma(0.95) < 10. Thus t_.95 _= 10 is a conservative estimate throughout the parameter space, for all cancer sites considered here, and the choice of N_10 _is appropriate (see Methods).

Table 4 shows Profile Likelihood 95% confidence intervals for ERR_1 _and ERR_0.025 _at optimal latency ϕ_m _as computed with the various estimated critical values.

**Table 4:** Profile Likelihood 95% confidence intervals for ERR at estimated critical values of

site	**ϕ**_**m**_	**t**_**.95**_	**ERR**_**1**_	**ERR**_**0.025**_		**ERR**_**1**_	**ERR**_**0.025**_		**ERR**_**1**_	**ERR**_**0.025**_
stom	11.89	10	(0.08,1.02)	(0.06,0.93)	9.72	(0.08,1.01)	(0.06,0.92)	8.16	(0.11,0.95)	(0.08,0.87)
liver	36.90	10	(0.38,3.21)	(0.19,2.59)	8.59	(0.44,3.05)	(0.23,2.45)	8.28	(0.46,3.01)	(0.25,2.42)
lung	13.60	10	(0.07,3.89)	(0.08,3.87)	9.44	(0.08,3.75)	(0.10,3.74)	8.48	(0.11,3.52)	(0.12,3.50)
panc	11.86	10	(0.48,-)	(0.38,-)	9.03	(0.60,-)	(0.49,-)	8.79	(0.64,-)	(0.52,-)
leuk	23.66	10	(-0.21,8.20)	(0.03,6.46)	7.93	(-0.09,7.05)	(0.06,5.51)	7.58	(-0.07,6.86)	(0.06,5.35)

t_.95_,  and : conservative, global and refined estimates of the 95% critical value of LRT for the hypothesis H_0_: (β, σ, τ) = (β_0_,σ_0_,τ_0_) vs. the alternative (β, σ, τ) ≠ (β_0_,σ_0_,τ_0_). See Methods.

Likewise from Additional File 1, Table **S4 **and using the notation in that file, all simulations of the null distribution of LRT_2p-lin _showed qlrt2(0.95) < 8 and qgamma2(0.95) < 8 so T_.95 _= 8 is a conservative estimate throughout the parameter space, for all cancer sites considered here. For any given site  is the supremum of qlrt2(0.95) and qgamma2(0.95) over simulations for that site.  is the supremum of qgamma2(0.95) over simulations for that site. Likewise  and  are the suprema of qgamma2(0.99) and qgamma2(0.999) over simulations for that site. Table 5 shows these critical values and the observed value of LRT_2p-lin _(see Table 2) for each site.

**Table 5:** Critical values and observed value of LRT_2p-lin_

Site	**ϕ**_**m**_	**T**_**.95**_					**LRT**_**2p-lin**_
Stom	11.89	8	7.542	7.276	10.394	14.708	19.64
Liver	36.90	8	6.779	6.603	9.329	13.082	27.64
Lung	13.60	8	7.418	6.930	9.795	13.740	14.74
Panc	11.86	8	7.372	7.007	10.049	14.265	14.80
Leuk	23.66	8	6.269	5.832	8.507	12.245	25.29

T_.95_, , : conservative, global and refined estimates of the 95% critical value of LRT for comparison of two-phase and linear models. See Methods.

, : refined estimates of the 99% and 99.9% critical value of LRT for comparison of two-phase and linear models. See Methods.

LRT_2p-lin _: observed value of LRT for comparison of two-phase and linear models. See Table 2.

Null distributions of  and LRT_2p-lin _for the stomach with ϕ = 5, 10,... 40 show little variation with latency and are similar to those obtained at ϕ_m _as shown in Table 6.

**Table 6:** LRT simulations for the stomach with (β_0_,σ_0_,τ_0_) = (0, 0, arbitrary) and various latencies

ϕ	N	qlrt2pcon	s	r	qgam2pcon	qlrt2plin	sl	rl	qgam2plin
5	500	9.53	2.39 (0.14)	0.59 (0.04)	9.26	7.72	2.07 (0.12)	0.66 (0.04)	7.39

10	500	8.79	2.56 (0.15)	0.65 (0.04)	8.67	6.87	2.09 (0.12)	0.70 (0.05)	7.02

15	500	8.90	2.46 (0.15)	0.64 (0.04)	8.59	6.89	2.02 (0.12)	0.72 (0.05)	6.63

20	500	8.21	2.38 (0.14)	0.63 (0.04)	8.48	6.87	2.00 (0.12)	0.72 (0.05)	6.60

25	500	8.70	2.62 (0.16)	0.65 (0.04)	8.84	7.58	2.01 (0.12)	0.66 (0.04)	7.20

30	500	8.51	2.31 (0.14)	0.63 (0.04)	8.28	6.71	1.84 (0.11)	0.68 (0.05)	6.62

35	500	8.84	2.31 (0.14)	0.61 (0.04)	8.54	7.09	1.79 (0.10)	0.65 (0.04)	6.79

40	500	8.46	2.45 (0.15)	0.67 (0.04)	8.11	6.95	1.86 (0.11)	0.70 (0.05)	6.46

11.89	1000	8.88	2.42 (0.10)	0.60 (0.03)	8.96	7.18	1.94 (0.08)	0.65 (0.03)	7.10

ϕ: latency

N: number of simulations

qlrt2pcon: 95^th ^percentile of

s, r: shape and rate parameters with their estimated standard errors for the fitted gamma distribution for

qgam2pcon: 95^th ^percentile of the fitted gamma distribution for

qlrt2plin: 95^th ^percentile of

sl, rl: shape and rate parameters with their estimated standard errors for the fitted gamma distribution for

qgam2plin: 95^th ^percentile of the fitted gamma distribution for

Simulation of the optimal latency  as described earlier also has implications for non-linearity. Again restricting to integral latencies, for the stomach LRT_2p-lin _> 9.4 when 5 ≤ ϕ ≤ 21 and 196 of 200 simulated datasets gave  in this range. For the liver LRT_2p-lin _> 16.7 when 34 ≤ ϕ ≤ 38 (198 of 200 simulations). For the lung LRT_2p-lin _> 7.7 when 7 ≤ ϕ ≤ 21 (184 of 200 simulations). For leukaemia LRT_2p-lin _> 9.4 when 17 ≤ ϕ ≤ 36 (191 of 200 simulations). Each of these results shows non-linearity (see Table 5) and for the stomach and liver the evidence is very strong. For the pancreas, LRT_2p-lin _> 9.5 when 7 ≤ ϕ ≤ 11 but only 156 of 200 simulations give  in this range.

The covariate "city" has little impact on the estimates of ERR when taken as an additional control. For the liver including "city", BC_a _95%CIs for ERR_1 _and ERR_0.025 _are strictly positive when 29 ≤ ϕ ≤ 43, again restricting to integral latencies. With "city" included in both models LRT_2p-con _attains its maximum subject to ERR_1 _≥ 0 when ϕ_m _= 37 and LRT_2p-con _= 28.41. At ϕ_m _= 37, ERR_1 _= 1.26 with BC_a _95%CI (0.61, 2.14) and ERR_0.025 _= 0.68 with BC_a _95%CI (0.18, 1.25). For comparison, without "city" the optimal integral latency is ϕ_m _= 37, at which ERR_1 _= 1.22 with BC_a _95%CI (0.59, 2.11) and ERR_0.025 _= 0.74 with BC_a _95%CI (0.23, 1.43). As a test of non-linearity (with "city" included in both models) LRT_2p-lin _> 6.8 when 29 ≤ ϕ ≤ 41, and LRT_2p-lin _> 18.15 when 34 ≤ ϕ ≤ 38, whereas without "city" LRT_2p-lin _> 6.86 when 32 ≤ ϕ ≤ 41 and LRT_2p-lin _> 16.73 when 34 ≤ ϕ ≤ 38.

Table 7 includes results with "city" for the 0 - 20 mSv dose range, for all 5 cancers. It also shows that fitting the two-phase model with "city" to the 0 - 20 mSv and 5 - 500 mSv dose ranges gives comparable results for ERR_1 _and latency, for the stomach, liver, lung, and leukaemia.

**Table 7:** Optimal (integral) latency, LRT and ERR, including "city", by dose range

Dose range	Site	**Φ**_**m**_	**LRT**_**2p-con**_ **at Φ**_**m**_	**LRT**_**2p-lin**_ **at Φ**_**m**_	**ERR**_**1**_ **at Φ**_**m**_	Latency Range	**ERR**_**0.025**_ **at Φ**_**m**_	Latency Range
0-20	Stom	11	13.02 p = 0.007	12.27 p = 0.003	0.38 (0.11, 0.70) (0.16, 0.65)*	5-18 5-21* 9-21^nl^	0.34 (0.11, 0.63) (0.14, 0.59)*	5-21 5-21*

5-500	Stom	8	9.03 p = 0.025	5.74 p = 0.07	0.29 (0.06, 0.60) (0.09, 0.55)*	7-9 5-11*		

0-20	Liver	37	28.41 p < 10^-7^	18.96 p < 10^-4^	1.26 (0.61, 2.14) (0.69, 1.97)*	29-43 29-43* 29-41^nl^	0.68 (0.18, 1.25) (0.24, 1.20)*	29-44 29-44*

5-500	Liver	37	14.47 p = 0.003	10.55 p = 0.005	0.74 (0.25, 1.43) (0.32, 1.30)*	34-41 34-42* 34-41^nl^		

0-20	Lung	16	10.43 p = 0.03	7.13 p = 0.05	1.00 (0.36, 1.84) (0.42, 1.72)*	10-21 10-21* 12-16^nl^	0.80 (0.21, 1.66) (0.28, 1.53)*	10-16 10-21*

5-500	Lung	16	21.69 p < 10^-4^	19.63 p < 10^-5^	1.33 (0.67, 2.22) (0.73, 2.10)*	7-21 7-21* 12-21^nl^		

0-20	Panc	10	14.89 p = 0.002	13.7 p < 0.001	11.32 (5.35, 16.46) (5.44, 15.60)*	5-11 5-11* 7-11^nl^	10.77 (4.21, 16.05) (4.70, 15.31)*	5-11 5-11*

5-500	Panc	42	1.97 p = 0.64	1.96 p = 0.38	0.44 (-, -)	-		

0-20	Leuk	25	21.66 p < 10^-4^	21.66 p < 10^-5^	1.39 (0.09, 4.31) (0.26, 3.79)*	24-25 24-26* 17-36^nl^	0.89 (0.11, 2.22) (0.16, 2.16)*	19-33 17-33*

5-500	Leuk	28	14.76 p < 0.001	8.60 p = 0.013	1.83 (0.45, 3.92) (0.59, 3.66)*	24-31 24-32* 24-28^nl^		

Dose range: data on which the two-phase model is fitted, including "city" as a control covariate

Φ_m_: latency, restricted to integers 5, 6,... 44, which maximises LRT_2p-con _subject to ERR_1 _≥ 0

LRT_2p-con_: Likelihood Ratio Test comparison of two-phase and control models at Φ_m. _p values derived from Table 8

LRT_2p-lin_: Likelihood Ratio Test comparison of two-phase and linear models at Φ_m. _p values derived from Table 8

ERR_1_: Excess Relative Risk at 10 mSv, from the two-phase model fitted across the dose range with latency Φ_m_, and its BC_a _95%CI (5000 replications)

Latency range: integral latency range around Φ_m _for which BC_a _95%CI is strictly positive

*: BC_a _90%CIs (5000 replications)

^nl^: integral latency range around Φ_m _for which LRT_2p-lin _> 6

ERR_0.025_: as above, for 0.25 mSv (computed only when Dose range = 0-20 mSv)

A limited analysis by simulation of LRT for the model with "city" was carried out over the 0 - 20 mSv and 5 - 500 mSv dose ranges, focused at the optimal (integral) latencies on the null distributions of LRT_2p-con _and LRT_2p-lin _at (β_0_,σ_0_,τ_0_) = (0, 0, arbitrary) and (β_0_,σ_0_,τ_0_) = (, 0, arbitrary) where  is obtained by fitting the linear model. Results are shown in Table 8 and the fitted gamma distributions are used to assign p-values to the observed LRT in Table 7.

**Table 8:** simulated null distribution of LRT_2p-con _and LRT_2p-lin _including "city", by dose range

Dose range	Site	**Φ**_**m**_	**LRT**_**2p-con**_ Shape	**LRT**_**2p-con**_ Rate	***t***_***0.95***_	**LRT**_**2p-lin**_ shape	**LRT**_**2p-lin**_ rate	***T***_***0.95***_
0-20	Stom	11	2.38	0.59	9.06	1.98	0.65	7.25
5-500	Stom	8	2.03	0.62	7.72	1.30	0.55	6.42
0-20	Liver	37	2.50	0.73	7.63	1.69	0.64	6.58
5-500	Liver	37	1.62	0.49	8.39	1.14	0.53	6.16
0-20	Lung	16	2.47	0.58	9.43	2.09	0.68	7.20
5-500	Lung	16	1.81	0.61	7.26	1.38	0.66	5.58
0-20	Panc	10	2.35	0.61	8.65	2.13	0.71	6.93
5-500	Panc	42	1.72	0.52	8.18	1.18	0.60	5.61
0-20	Leuk	25	2.11	0.62	7.94	1.59	0.67	6.01
5-500	Leuk	28	1.87	0.62	7.34	1.25	0.56	6.14

gamma distributions are fitted to 500 simulations of LRT_2p-con _for the null hypothesis (β_0_,σ_0_,τ_0_) = (0,0, arbitrary) and of LRT_2p-lin _for the null hypothesis (β_0_,σ_0_,τ_0_) = (,0, arbitrary) where  is the fitted parameter for the linear model. Models include "city" as a control covariate and are fitted at the optimal (integral) latency Φ_m _for the site and dose range.

shape: shape parameter of the fitted gamma distribution

rate: rate parameter of the fitted gamma distribution

*t*_*0.95*_: critical value of the fitted gamma distribution for

*T*_*0.95*_: critical value of the fitted gamma distribution for

From Table 8 (with "city") and Additional File 1, Table **S4 **(without "city"), the criterion LRT_2p-lin _> 6 is a test of non-linearity at 90% level. Accordingly, Table 7 also shows the latency ranges surrounding Φ_m _for which LRT_2p-lin _> 6.

Other aspects of the analysis (simulated null distributions of LRT_2p-con _and LRT_2p-lin _across the parameter space and simulated variation in optimal latency ϕ_m_) were not repeated with "city".

For the stomach, liver, and lung Table 9 shows the results when the two-phase model with "city" is fitted separately to the male and female 0 - 20 mSv subcohorts. For the pancreas and leukaemia, there were too few cases to split the data by gender.

**Table 9:** Male and Female subcohorts, with "city", 0-20 mSv, integral latency

Sex	Site	**Φ**_**mabs**_	**Φ**_**m**_	**LRT**_**2p-con**_ **at Φ**_**m**_	**LRT**_**2p-lin**_ **at Φ**_**m**_	**ERR**_1_ **at Φ**_**m**_	Latency Range	**ERR**_**0.025**_ **at Φ**_**m**_	Latency Range
M	Stom	6	6	11.82	11.06	2.41 (1.24-3.52)	5-6 5-6^nl^	2.30 (0.99-3.47)	5-6

F	Stom	10	10	12.26	10.67	0.57 (0.18-1.10)	9-13 9-18^nl^	0.39 (0.07-0.56)	7-13

M	Liver	37	37	19.40	14.19	1.55 (0.58-3.05)	34-42 34-38^nl^	1.00 (0.14-1.58)	34-42

F	Liver	20	36	10.90	8.82	1.01 (0.05-1.88)	34-38 27-36^nl^	0.86 (0.04-1.38)	27-38

M	Lung	37	16	6.04	4.92	0.89 (0.16-2.00)	12-16	0.66 (0.09-1.93)	12-16

F	Lung	21	21	7.44	6.17	1.18 (0.41-2.28)	14-21 19-21^nl^	0.95 (0.27-1.57)	14-21

notation as in Table 7

Table 10 shows the linear model with "city" fitted to the 0 - 20 mSv, 0 - 5 mSv, 5 - 20 mSv, 0 - 0.5 mSv, and 0.5 - 20 mSv subcohorts for the liver and lung.

**Table 10:** Linear model with "city" for liver and lung on partitions of 0-20 mSv, by latency.

	Liver										Lung									
range	0-20 mSv		0-5 mSv		5-20 mSv		0-0.5 mSv		0.5-20 mSv		0-20 mSv		0-5 mSv		5-20 mSv		0-0.5 mSv		0.5-20 mSv	
p-y	1690392		1152356		538039		1141938		548454		1690392		1152356		538039		1141938		548454	
ϕ	LRT	β	LRT	β	LRT	β	LRT	β	LRT	β	LRT	β	LRT	β	LRT	β	LRT	β	LRT	β
6	0.01	-0.01	0.52	6.22	0.28	3.70	1.20	12.63	0.11	0.14	1.43	0.13	0.00	0.00	0.01	-0.14	0.00	0.00	0.17	0.18
7	0.02	-0.02	0.30	-2.00	0.05	-0.11	0.53	-4.57	0.00	0.01	1.40	0.13	0.10	2.23	0.00	-0.03	1.13	12.62	0.18	0.16
8	0.02	-0.02	0.30	-2.00	0.05	-0.11	0.53	-4.57	0.00	0.01	1.40	0.13	0.10	2.23	0.00	-0.03	1.13	12.62	0.18	0.16
9	0.01	-0.01	0.29	-2.00	0.01	-0.05	0.60	-4.71	0.00	0.02	1.29	0.12	0.00	0.00	0.08	-0.16	0.53	7.48	0.09	0.11
10	0.76	-0.08	1.02	-2.00	7.09	-0.50	3.04	-9.15	5.00	-0.46	1.63	0.14	0.13	2.45	0.35	0.37	1.66	14.30	0.69	0.31
11	0.75	-0.08	0.99	-2.00	7.06	-0.50	3.02	-9.12	4.96	-0.46	1.64	0.14	0.13	2.51	0.37	0.38	1.68	14.39	0.71	0.31
12	0.80	-0.09	1.18	-2.00	6.77	-0.50	3.81	-9.81	5.60	-0.47	2.15	0.16	0.81	6.90	1.98	0.95	3.55	22.14	2.11	0.57
13	0.79	-0.09	1.28	-2.00	6.70	-0.50	4.33	-10.24	5.48	-0.46	2.15	0.16	0.81	6.93	1.99	0.95	3.57	22.20	2.11	0.57
14	1.10	-0.10	2.52	-2.00	8.76	-0.50	14.14	-14.71	7.80	-0.50	3.29	0.20	0.07	1.69	7.75	2.31	2.42	14.89	6.46	1.15
15	1.10	-0.10	2.52	-2.00	8.40	-0.50	14.14	-14.71	7.83	-0.50	3.29	0.20	0.07	1.69	7.75	2.31	2.42	14.89	6.46	1.15
16	1.09	-0.10	2.49	-2.00	8.65	-0.50	14.08	-14.70	7.76	-0.50	3.30	0.20	0.08	1.77	7.81	2.32	2.45	15.00	6.51	1.15
17	0.94	-0.10	2.23	-2.00	8.11	-0.50	12.11	-14.38	7.63	-0.50	1.97	0.16	0.12	2.10	1.37	0.53	2.43	14.12	2.14	0.51
18	0.92	-0.10	2.21	-2.00	8.93	-0.56	11.96	-14.33	7.46	-0.51	1.98	0.16	0.12	2.15	1.40	0.54	2.46	14.24	2.17	0.52
19	0.89	-0.10	2.33	-2.00	9.31	-0.56	10.63	-14.12	8.27	-0.52	1.70	0.15	1.69	9.36	0.75	0.34	5.17	21.98	1.74	0.44
20	0.89	-0.10	2.33	-2.00	9.30	-0.56	10.63	-14.12	8.27	-0.52	1.70	0.15	1.69	9.36	0.75	0.34	5.17	21.98	1.74	0.44
21	0.88	-0.10	2.31	-2.00	9.86	-0.59	10.56	-14.10	8.20	-0.52	1.72	0.15	1.74	9.53	0.78	0.35	5.23	22.17	1.77	0.44
22	0.32	-0.06	1.75	-2.00	5.51	-0.50	7.84	-13.34	4.73	-0.44	0.68	0.09	0.04	1.04	0.18	-0.13	0.41	4.53	0.08	0.08
23	0.25	-0.05	1.89	-2.00	4.52	-0.47	8.88	-13.81	3.96	-0.41	0.58	0.09	0.05	1.15	0.40	-0.19	0.44	4.72	0.02	0.04
24	0.02	0.02	0.99	-2.00	0.49	-0.19	5.71	-12.83	0.52	-0.18	0.36	0.07	0.00	0.00	0.66	-0.22	0.22	3.13	0.00	0.00
25	0.02	0.02	0.99	-2.00	0.48	-0.19	5.71	-12.83	0.52	-0.18	0.36	0.07	0.00	0.00	0.66	-0.22	0.22	3.13	0.00	0.00
26	0.02	0.02	1.08	-2.00	0.46	-0.19	6.00	-13.00	0.50	-0.17	0.37	0.07	0.00	0.00	0.63	-0.22	0.23	3.25	0.00	0.00
27	0.38	0.08	0.27	-2.00	0.08	0.09	2.54	-9.59	0.03	0.05	0.35	0.07	0.82	-2.00	0.36	-0.17	0.29	-3.18	0.02	0.04
28	0.35	0.07	0.40	-2.00	0.07	0.08	2.89	-10.05	0.02	0.04	0.44	0.08	0.80	-2.00	0.16	-0.12	0.25	-2.98	0.12	0.10
29	1.18	0.15	0.00	0.00	1.29	0.40	0.41	-4.55	0.92	0.30	0.15	0.05	0.32	-2.00	0.68	-0.21	0.23	3.18	0.01	-0.02
30	1.19	0.15	0.00	0.00	1.30	0.40	0.41	-4.55	0.93	0.30	0.15	0.05	0.32	-2.00	0.67	-0.21	0.23	3.18	0.01	-0.02
31	1.21	0.15	0.00	0.00	1.35	0.41	0.39	-4.43	0.97	0.31	0.16	0.05	0.29	-2.00	0.63	-0.20	0.25	3.27	0.01	-0.02
32	1.42	0.17	0.00	0.00	1.27	0.38	0.16	3.36	0.85	0.28	0.14	0.05	0.40	-2.00	0.39	-0.16	0.00	0.00	0.00	0.01
33	1.29	0.16	0.00	0.00	1.10	0.35	0.19	3.61	0.71	0.25	0.09	0.04	0.35	-2.00	0.60	-0.19	0.00	0.00	0.01	-0.02
34	5.02	0.37	4.32	18.20	7.44	1.08	6.53	26.15	6.28	0.90	0.08	-0.04	0.24	-2.00	2.25	-0.31	0.00	0.00	0.84	-0.20
35	5.02	0.37	4.32	18.20	7.46	1.08	6.53	26.15	6.30	0.90	0.08	-0.04	0.24	-2.00	2.24	-0.31	0.00	0.00	0.84	-0.20
36	5.08	0.38	4.45	18.55	7.56	1.09	6.63	26.40	6.39	0.91	0.07	-0.04	0.24	-2.00	2.17	-0.31	0.00	0.00	0.80	-0.19
37	9.45	0.58	2.86	13.80	14.18	1.68	4.48	19.51	12.57	1.45	0.22	-0.07	0.42	-2.00	2.15	-0.30	0.13	-1.96	1.01	-0.21
38	8.62	0.56	3.15	14.53	12.94	1.54	4.85	20.35	11.46	1.33	0.16	-0.06	0.37	-2.00	1.84	-0.28	0.00	0.00	0.80	-0.19
39	9.61	0.71	1.20	8.11	10.39	1.18	1.28	9.11	9.75	1.09	0.26	-0.09	0.00	0.00	1.23	-0.24	0.13	2.12	0.64	-0.18
40	9.61	0.71	1.20	8.11	10.39	1.18	1.28	9.11	9.75	1.09	0.26	-0.09	0.00	0.00	1.23	-0.24	0.13	2.12	0.64	-0.18
41	9.74	0.71	1.28	8.39	10.56	1.19	1.33	9.33	9.91	1.11	0.24	-0.09	0.00	0.00	1.17	-0.23	0.14	2.24	0.60	-0.17
42	5.17	0.62	0.06	1.85	3.96	0.65	0.03	1.29	3.78	0.62	0.96	-0.22	1.12	6.72	2.12	-0.34	0.83	6.09	1.52	-0.29
43	4.18	0.55	0.07	1.93	2.96	0.55	0.03	1.29	2.81	0.53	0.92	-0.21	1.15	6.82	2.05	-0.33	0.83	6.09	1.45	-0.29
44	1.48	0.47	1.08	10.34	0.25	0.18	1.26	11.40	0.28	0.19	0.53	-0.25	0.15	3.14	0.85	-0.32	0.00	0.00	0.67	-0.29

range: dose range over which the linear model is fitted

p-y: person-years for the dose range

LRT: LRT_lin-con _when the linear model is fitted at latency ϕ. LRT > 3.84 indicates significance at p < 0.05

β: fitted parameter value.

## Discussion

This paper develops and partially corrects the approach in [2] and the two should be read in conjunction. I focus initially on the 0 - 20 mSv dose range, which provides over 60% of the person-years of observation in the Life Span Study 12 cohort. Over the full data range 0 - 8000 mSv and the truncated range 0 - 4000 mSv the dose response for cancer mortality is known to be approximately linear and risk estimates for 1000 mSv have been obtained through the ongoing Life Span Study project. The ICRP recommendations for radiation protection are then based on linear extrapolation, as the Excess Relative Risk from 10 mSv is presumed to be 0.01 times the ERR from 1000 mSv.

If we were confident that the approximate linearity of the dose response extends to the low dose region, then linear extrapolation would be justified. But this is not known, and the response may be approximately linear over a wide dose range but highly non-linear at lower doses. There are many examples of non-linear dose response in radiation cell biology [16,17].

Thus, if the Japanese data is used to derive risk estimates for doses such as 10 mSv or 1mSv which are directly relevant to occupational and public exposure, it makes sense to consider the 0 - 20 mSv data in its own right and in relation to wider dose ranges, rather than to pool the data and apply linear extrapolation.

Secondly, a latency parameter ϕ is used to assess the delay between exposure and cancer mortality. Although it would be better to allow latency to modify dose by some smooth function rather than an abrupt switch from no effect to full effect when Time-Since-Exposure passes ϕ, this is much harder to compute and was not attempted here.

**Figure 6:**
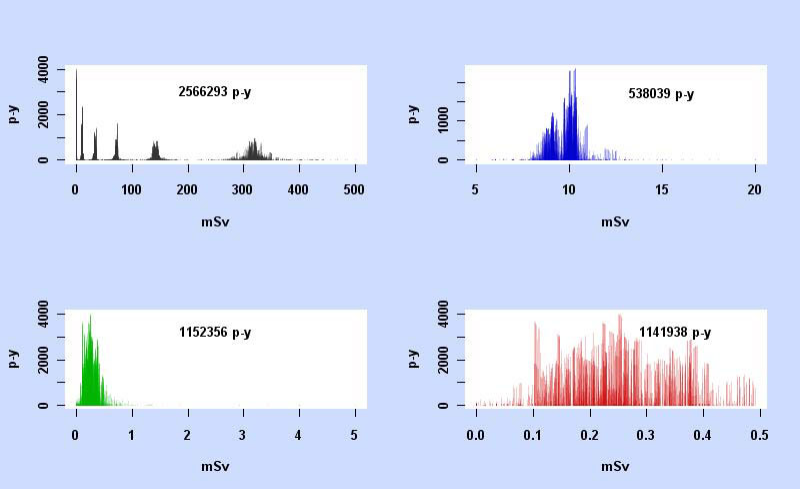
**p-y observation against mean dose**. For the subcohorts 0-500 mSv, 5-20 mSv, 0-5 mSv, and 0-0.5 mSv, each data cell in the subcohort is represented by a vertical line at its mean dose, with height the p-y observation in the data cell. The total p-y observation for each subcohort is also shown.

As pointed out during peer review, we should consider at the outset whether the data is sufficient for fitting a non-linear model based on mean dose. Figure 6 shows scatterplots of p-y observation against mean dose, for the ranges 0 - 500 mSv, 5 - 20 mSv, 0 - 5 mSv, and 0 - 0.5 mSv. Clearly the 0 - 500 mSv and 5 - 500 mSv data contains a wide variety of doses. The 5 - 20 mSv data shows a spread of mean doses. Whilst the 0 - 5 mSv data is concentrated below 1 mSv, there is a spread of mean doses in that region. Each dose range determines a very large dataset.

The fact that for the stomach, liver, lung, and leukaemia rather similar results for ERR_1 _and latency are obtained from fitting the two-phase model across the 5 - 500 mSv dose range as from fitting across the 0 - 20 mSv dose range suggests that there is also sufficient information in the 0 - 20 mSv data to justify fitting this model. The stomach, with the largest number of cases, shows fairly tight confidence intervals for Excess Relative Risk over a broad range of latencies, which supports the validity of the model.

If the dose response were linear, similar estimates of its slope (β) should be obtained over different dose ranges. As the liver and lung data illustrate (Table 10), fitting the linear model separately to subcohorts of the 0 - 20 mSv dose range yields very different estimates of the dose response. For the liver, there are significant results at comparable latencies in the 0 - 20 mSv dose range and all the subranges, but the estimates of β from doses below 5 mSv (or below 0.5 mSv) are completely different from those obtained from doses above 5 mSv (or 0.5 mSv), in fact around 20 times higher when latency = 36 years. For the lung, the linear model detects some significant activity in restricted dose ranges, but the results are very different, and combining the two dose ranges yields insignificant results. By contrast, see Table 7, the two-phase model shows significant results for the lung with the 0 - 20 mSv data for latencies from 10 to 21 years.

**Figure 7:**
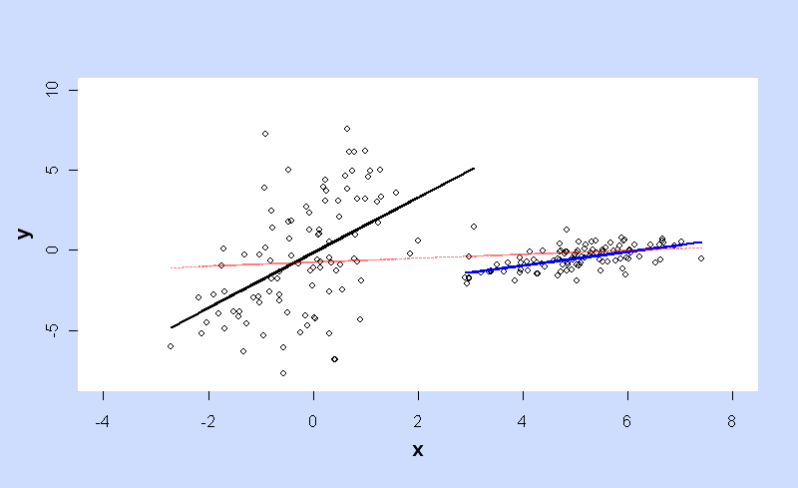
**Ordinary Least Squares fitting to (synthetic) univariate data with a bimodal independent variable**. Regression of y on x is significantly positive over portions of data (black and blue lines) but lacks significance over the full range (red line).

It is not unusual for a model to achieve significance on portions of the data but fail to do so when they are combined. Even univariate Ordinary Least Squares regression on a bimodal independent variable may illustrate this, as in Figure 7 with synthetic data.

As discussed in [2] the graphs of ERR against latency show a clear separation into positive and negative regions, as is also apparent with the linear model over restricted dose ranges for the liver and lung. For any given model, the "optimal latency" ϕ_m _identifies the time lag which maximises LRT within the region of non-negative ERR_1_. The existence of a significant raised risk is of interest whether or not other latencies show significant decrease in risk, with possibly higher LRT. Frequently ϕ_m _coincides with ϕ_mabs _which maximises LRT without constraint, but the distinction is relevant for the lung (Table 2) and more generally when simulating the variation in latency.

The point estimates of optimal latency ϕ_m _and ERR obtained previously are confirmed by Table 2. The next stage of analysis concerns confidence intervals for ERR at the optimal latency. Statistical inference in [2] was based on the Likelihood Ratio Test but it was wrongly assumed, as Little [3] pointed out, that Wilks theorem held for the non-linear models so that LRT would be asymptotically χ^2 ^distributed, if the null hypothesis (specifying parameters in the model) were true.

One of the regularity conditions required for Wilks Theorem is that distinct values of the model parameters produce distinct probability distributions. In the two-phase model when σ = 0, ERR = βD_ϕ _irrespective of the value of τ. The asymptotic properties of models with an indeterminate parameter (which cannot be identified from the distribution) have been analysed [18-21] but I was unable to use these methods and therefore took an intensive computational approach, which applies to the given set of records rather than asymptotically.

The parametric bootstrap is an established technique for finding confidence intervals in the absence of any information on the distribution of LRT. The percentile method refits the model to simulated data generated by samples from the model whose parameter values were obtained by fitting to the actual data. The BC_a _method adjusts for the fact that simulation may produce biased estimates of the statistic of interest (in this case ERR) and of its variance. Both methods gave good coverage with almost identical rates when tested on the stomach data, and comparable confidence intervals for all the sites analysed. The BC_a _method is much faster and generally preferable.

However, the underlying assumption of the parametric bootstrap is open to question. Perhaps the observed data arose from sampling at parameter values other than those obtained by fitting the model to that data. Confidence Intervals based on LRT avoid this assumption but depend on the critical value t_.95 _used to reject the null hypothesis if LRT > t_.95_.

If Wilks Theorem had applied, then LRT for testing H_0_: (β, σ, τ) = (β_0_,σ_0_,τ_0_) vs. the alternative (β, σ, τ) ≠ (β_0_,σ_0_,τ_0_) would have the asymptotic distribution LRT ~ χ^2 ^on 3 d.f. if H_0 _holds, for *any *H_0_. Thus t_.95 _= F^-1^(0.95) = 7.8147 where F is the cumulative χ^2 ^on 3 d.f. would be the appropriate critical value, rejecting H_0 _if LRT > t_.95_.

**Figure 8:**
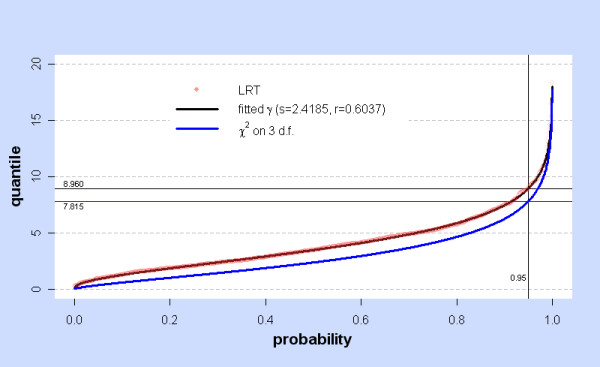
**Simulated LRT at H_0_: (β, σ, τ) = (0,0,τ_0_) with fitted γ and χ^2^: stomach**. At a given probability value, the red dot shows the corresponding quantile of the simulated values (N = 1000) of LRT =  for the stomach with the null hypothesis H_0_: (β_0_,σ_0_,τ_0_) = (0,0, arbitrary). For this H_0_,  = LRT_2p-con_. The black line shows quantiles of the fitted gamma distribution γ, with shape s and rate r. The blue line shows quantiles of the χ^2 ^(3 d.f.) distribution. The horizontal lines marked 8.960 and 7.815 show the 0.95 quantiles of γ and χ^2 ^(3 d.f.).

In the absence of any theoretical prediction, the null distribution of LRT can still be estimated by Monte Carlo simulation but may depend on the choice of H_0 _as seen in Additional File 1, Tables **S1, S2, S3 **and **S4**. In fact Additional File 1, Tables **S2 **and **S3 **show the null distribution of  departs from χ^2 ^on 3 d.f. as σ_0 _→ 0, while Additional File 1, Table **S4 **shows that the null distribution of LRT_2p-lin _departs from χ^2 ^on 2 d.f. everywhere. Table 3 indicates that the simulated null distribution of  is fairly independent of the parameters for the control covariates, and depends only on H_0_: (β, σ, τ) = (β_0_,σ_0_,τ_0_). The null distributions when σ_0 _= 0 are quite different from those when σ_0 _>> 0, as expected since it is σ_0 _= 0 which causes the indeterminacy of the model and the failure of Wilks Theorem on this hyperplane. Gamma distributions give a reasonable fit to the simulated null distribution of  in general (see Additional File 1, Tables **S1, S2 **and **S3**). When H_0 _has σ_0 _= 0, typical shape and rate parameters are s ~ 2.24 and r ~ 0.63, while for σ_0 _>> 0 s ~ 1.5 and r ~ 0.5 as predicted by Wilks Theorem. Figure 8, the probablity plot for the stomach with (β_0_,σ_0_,τ_0_) = (0,0, arbitrary) shows the failure of Wilks Theorem and the close fit of simulated  = LRT_2p-con _with a gamma distribution (s = 2.418, r = 0.604).

**Figure 9:**
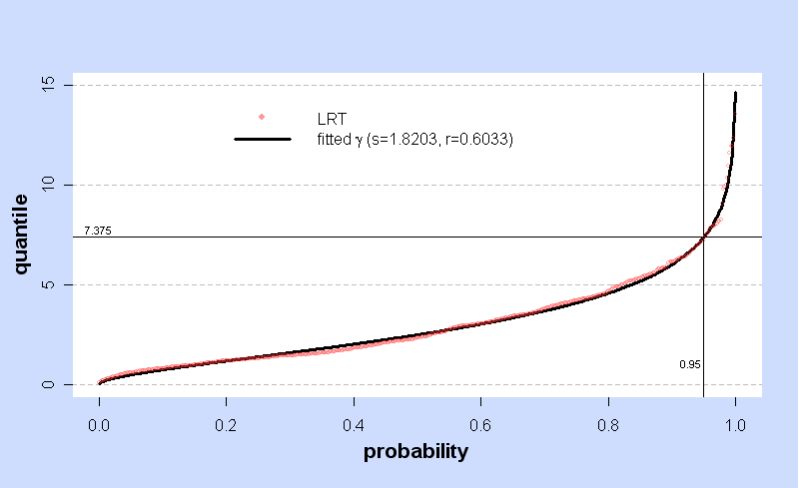
**Simulated LRT at H_0_: (β, σ, τ) = (7.297,0,τ_0_) with fitted γ: liver**. At a given probability value, the red dot shows the corresponding quantile of the simulated values (N = 500) of LRT =  for the liver with the null hypothesis H_0_: (β_0_,σ_0_,τ_0_) = (7.297,0, arbitrary). The black line shows quantiles of the fitted gamma distribution γ, with shape s and rate r. The horizontal line marked 7.375 shows the 0.95 quantile of γ.

The Kolmogorov-Smirnov test against the fitted distribution is extremely sensitive and even when it distinguishes LRT from the fitted gamma, a probability plot may show close agreement. For example for the liver, with (β_0_,σ_0_,τ_0_) = (7.297, 0, arbitrary), the K-S test has D = 0.058 with simulated p = 0.001 but the plot (Figure 9) is fairly close.

Likewise, fitted gamma distributions generally give a reasonable fit to the null distribution of LRT_2p-lin _with typical parameters s ~ 1.79, r ~ 0.68.

For the linear model, Additional File 1, Table **S4 **shows simulation of the null distribution of  generally conforms to a χ^2 ^distribution on 1 d.f. as expected from Wilks theorem. However as β_0 _→ -0.5, the null distribution approaches a γ distribution with shape = 0.5, rate = 1. Since β_0 _= -0.5 specifies a boundary of the parameter space, the theory of mixture models [22] predicts, at the boundary, the null distribution of  ~ 1/2 χ^2 ^(1 d.f.) = 1/2 γ (0.5,0.5) = γ (0.5,1). The confidence intervals shown in Figure 2 for liver and urinary cancers involve β values far from the boundary and Wilks theorem is valid in this region.

For all simulations and all cancer sites (Additional File 1, Tables **S1, S2 **and **S3**) the 95^th ^percentile of the fitted gamma distributions and the 95^th ^percentile of the simulated  itself are all below 10. Additional File 1, Tables **S1, S2, and S3 **summarise nearly 50,000 simulations of LRT at a wide variety of randomly chosen values of H_0 _. On that basis, t_.95 _= 10 is chosen as a conservative estimate applying throughout the parameter space for all cancer sites considered here. H_0 _can be rejected whenever  > 10, i.e. whenever (β_0_,σ_0_,τ_0_) is outside the neighbourhood N_10 _of the fitted parameters (). N_10 _is thus an appropriate neighbourhood, as defined in Methods.

Inside this neighbourhood Additional File 1, Table **S1 **shows gamma distributions generally fit the simulated  very well, justifying the definition of  from fitted gamma distributions within N_10_. The intermediate choice  is based on simulations inside and outside N_10 _and uses both the simulations and the fitted distributions to adjust the critical value upwards as either may be underestimates.

For the stomach, liver, and lung, confidence intervals for ERR obtained by the bootstrap (Table 2) or from simulation of  (Table 4) are broadly similar, whichever critical value or bootstrap method is chosen. LRT intervals using  show closer agreement with the bootstrap methods. For these 3 cancers, all the LRT-based intervals for ERR_1 _and ERR_0.025 _exclude 0 as do the bootstrap intervals, which are tighter. This validates the claim in [2] that ERR_1 _is significantly elevated for the stomach, liver, and lung.

All five cancers show significant non-linearity of the dose response by LRT comparison of the two-phase and linear models (Table 5). For the stomach, liver, and leukaemia the two-phase model is preferable with p << 0.001, while for the lung and pancreas p < 0.01.

The previous discussion concerns behaviour at the optimal latency ϕ_m_, an unknown parameter estimated from the data. BC_a _intervals, comparable at ϕ_m _to CIs obtained by other methods, were used to investigate ERR across a range of latencies, restricted to integers 5, 6,... 44. Each cancer site has latency ranges in which BC_a _CIs for ERR_1 _and ERR_0.025 _are strictly positive. Figure 5 illustrates this for the stomach and also shows that ERR is significantly raised when the dose is not modified by latency (ϕ = 6 is below the minimum time-since-exposure in the 0 - 20 mSv cohort). With the linear model, liver and urinary cancers are significantly elevated over a range of long latencies, showing similar variation (Figures 1 and 2).

Fitting the two-phase model at ϕ_m_, simulating new data and finding the simulated optimal latency  gives an estimate of the probability that the true value of ϕ_m _lies in any given region. For the liver and stomach, this approach gives very strong evidence that ϕ_m _lies in the regions where ERR_1 _and ERR_0.025 _are strictly positive, and where the two-phase model is clearly superior (by LRT) to the linear model. For the lung, the evidence on these points is still valid, though weaker. For leukaemia, there is strong evidence that ϕ_m _lies in the region where the dose response is non-linear, and likewise in the region where the 95% CI for ERR_0.025 _is strictly positive. However, with the variation in ϕ_m _we cannot be confident that the 95% CI for ERR_1 _is strictly positive.

Many results for the pancreas are considerably weaker than for other sites. Even at the optimal latency, the confidence intervals are extremely wide. With the BC_a _method, the lower limit is attained as the minimum bootstrap value, and is assigned a probability > 0.025. The "bias" and "acceleration" of the BC_a _bootstrap sample are both large, unlike those for other sites. When the uncertainty in the optimal latency is determined by simulation at ϕ_m _we cannot be confident that the true optimal latency for the pancreas lies within the range where ERR > 0 or where non-linearity is detected by LRT_2p-lin_.

Up to this point, the primary aim was to test the validity of the conclusions in [2], and accordingly "city" was not included as a control covariate and the dose range remained at 0 - 20 mSv. A limited reanalysis including "city" in the baseline model and comparing the 0 - 20 mSv and 5 - 500 mSv dose ranges is shown in Tables 7 and 8. The higher dose range was chosen to include 10 mSv, but to exclude the lowest dose category in the LSS12 dataset. Above 5 mSv the revised dosimetry DS02 generally agrees with the DS86 dosimetry used here (and in [2]). Comparing these ranges tests whether the results from 0 - 20 mSv simply reflect some anomaly in the lowest dose category. With the exception of the pancreas, there is a striking similarity between results from the 0 - 20 mSv and 5 - 500 mSv dose ranges, which show comparable estimates of the optimal latency, latency ranges, and the Excess Relative Risk from 10 mSv.

I do not know if any other approach to baseline risk may explain these effects. However, significant non-linearity and positive ERR are also found for the stomach, liver and lung when the data is restricted to male or female subcohorts, with "city" included in the model (Table 9). Any possible interaction effects involving gender would disappear with the all-male or all-female data. As mentioned in [2] the use of log mean attained age was an adequate alternative to the full set of attained age categories, and alternative controls affected the estimates of ERR by no more than a factor of 2.

The results at 0.25 mSv (ERR_0.025_) show that the risk detected here arises at extremely low doses. If there are doubts about the accuracy of the DS86 dosimetry, the question remains as to why the DS86 dose is a significant predictor of risk.

Overall, the results have several possible interpretations. They may reflect confounding by other covariates not shown in the public dataset, which may be correlated with the external dose used here (and in LSS12) and may also interact with radiation and may vary over time. Differences between the rural and urban populations may contribute to the non-linear effects. Or, these may be caused by gross underestimates of the external dose (unlikely) or by internal doses arising, for example, from "black rain". Such interpretations would be specific to the Japanese cohort, and would therefore cast doubt on using this data to predict radiation response elsewhere.

However, the results may also indicate that low doses of radiation do impact on cancer mortality, provided that latency is included in the model and the dose response is not constrained to be linear. In radiation cell biology, non-linear dose response is known for the bystander effect using a variety of endpoints and the two-phase model was proposed as a simplified form of a model for the bystander effect [16].

This paper does not reconsider any of these possible interpretations [2]. However, I hope it establishes the statistical validity of this analysis of the available public data for the 1950-1990 mortality cohort.

## Conclusion

This reanalysis validates the main conclusions of [2]. Bootstrap methods and Monte Carlo simulation of LRT show that Excess Relative Risk for cancer mortality in Japanese A-bomb survivors exposed to external radiation doses below 20 mSv is positive, large, and significant for various cancers, as detected by models incorporating latency. The dose response is highly non-linear for the stomach, liver, lung, pancreas, and leukaemia. In each case the two-phase model shows large Excess Relative Risk at 10 mSv external dose lagged by the optimal latency for the cancer. LRT-based 95% Confidence Intervals are strictly positive, except for leukaemia. Bootstrap BC_a _95% CIs are strictly positive for all five cancers over a range of latencies. Large, positive Excess Relative Risk is also found when the male and female data is analysed separately for the stomach, liver and lung.

When the optimal latency varies by simulation, the stomach, liver, lung and leukaemia still show non-linear dose response, and likewise ERR > 0 at 95% level for the stomach, liver, lung.

With "city" included as a control covariate in the two-phase model, similar estimates of latency and ERR at 10 mSv are obtained for the stomach, liver, lung, and leukaemia whether the dose range is 0 - 20 mSv or 5 - 500 mSv. Dose response for the liver, lung, and leukaemia is significantly non-linear in the 5 - 500 mSv range, particularly for the lung. Such results cannot be explained by any anomaly of the 0 - 5 mSv data.

The linear model finds significant results for the liver and urinary tract over a range of long latencies.

This analysis of cancer mortality in Japanese A-bomb survivors exposed to low doses of external radiation in 1945 shows significant non-linearity of dose response and significant large Excess Relative Risk over a range of latencies. These findings do not support the current ICRP recommended annual occupational dose limit of 20 mSv.

## Abbreviations

BC_a_: Bias-corrected accelerated; CEDR: Comprehensive Epidemiological Data Resource; CI: Confidence Interval; DS02: Dosimetry System 2002; DS86: Dosimetry System 1986; ERR: Excess Relative Risk; ICRP: International Commission on Radiological Protection; K-S: Kolmogorov-Smirnov; LF: Least Favourable; LRT: Likelihood Ratio Test; LSS: Life Span Study; MLE: Maximum Likelihood Estimate; mSv: milliSievert; p-y: person-years; RERF: Radiation Effects Research Foundation.

## Competing interests

The author declares that they have no competing interests.

## Authors' contributions

GD conceived of and designed the study and carried out the statistical analysis.

## Supplementary Material

Additional file 1 Contains index file for downloading 4 Tables, 3 Code Files, 2 Commentary Files, and 5 Data Files. Click here for file

## References

[B1] ICRP 1990 Recommendations of the International Commission on Radiological Protection Publication 60, Annals of the ICRP 21 (1-3) 1991 Oxford 2053748

[B2] Dropkin G Low dose radiation and cancer in A-bomb survivors Environ Health 2007 6 1 10.1186/1476-069X-6-1 17233918 PMC1785370

[B3] Little Mark Statistical problems with analysis of Dropkin http://www.ehjournal.net/content/6/1/1/comments#260548

[B4] Pierce DA Shimizu Y Preston DL Vaeth M Mabuchi K Studies of the Mortality of Atomic bomb survivors. Report 12, Part 1, Cancer: 1950-1990 Radiat Res 1996 146 1 1 27 10.2307/3579391 8677290

[B5] Dudley R MIT OpenCourseWare | Mathematics | 18.466 Mathematical Statistics, Spring 2003 | Lecture Notes http://ocw.mit.edu/OcwWeb/Mathematics/18-466Mathematical-StatisticsSpring2003/LectureNotes/

[B6] Efron B Tibshirani RJ An Introduction to the Bootstrap 1993 New York: Chapman & Hall

[B7] Davison AC Hinkley DV Bootstrap Methods and their Application 1997 Cambridge: Cambridge University Press

[B8] R Development Core Team R: A language and environment for statistical computing 2007 Vienna, Austria: R Foundation for Statistical Computing http://www.R-project.org

[B9] RERF (Radiation Effects Research Foundation) http://www.rerf.or.jp/

[B10] CEDR (Comprehensive Epidemiological Data Resource) Data File Set JALSSA03 File ID: R12CANC http://cedr.lbl.gov/cgi-bin/spiface/find/cedrfile/?FILE=1040] accessed 19 September 2002

[B11] R source code based on Nash JC Compact Numerical Methods for Computers: Linear Algebra and Function Minimisation 1990 CRC Press

[B12] DiCiccio TJ Efron B Bootstrap Confidence Intervals Stat Sci 1996 11 3 189 228 http://projecteuclid.org/DPubS/Repository/1.0/Disseminate?view=body&id=pdf_1&handle=euclid.ss/1032280214 10.1214/ss/1032280214

[B13] Pinheiro JC Bates DM Mixed-effects models in S and S-PLUS 2000 New York: Springer

[B14] Venables WN Ripley BD Modern Applied Statistics with S 2002 4 New York: Springer

[B15] Geyer C Kolmogorov-Smirnov and Lilliefors Testslecture notes Statistics 5601, Univ of Minnesota http://www.stat.umn.edu/geyer/5601/examp/kolmogorov.html#one-test

[B16] Brenner DJ Little JB Sachs RK The bystander effect in radiation oncogenesis: II. A quantitiative model Radiat Res 2001 155 3 402 408 10.1667/0033-7587(2001)155[0402:TBEIRO]2.0.CO;2 11182790

[B17] Geras'kin SA Oudalova AA Kim JK Dikarev VG Dikareva NS Cytogenetic effect of low dose γ-radiation in *Hordeum vulgare *seedlings: non-linear dose-effect relationship Radiat Environ Biophys 2007 46 31 41 10.1007/s00411-006-0082-z 17171549

[B18] Davies RB Hypothesis Testing When a Nuisance Parameter is Present Only Under the Alternative Biometrika 1977 64 2 247 254 10.2307/2335690 15737101

[B19] Davies RB Hypothesis Testing When a Nuisance Parameter is Present Only Under the Alternatives Biometrika 1987 74 1 33 43 10.1093/biomet/74.1.33

[B20] Cheng RCH Traylor R Non-Regular Maximum Likelihood Problems J R Stat Soc Series B Stat Methodol 1995 57 1 3 44 http://www.jstor.org/pss/2346086

[B21] Andrews DWK Ploberger W Admissibility of the Likelihood Ratio Test When a Nuisance Parameter is Present Only Under the Alternative Ann Stat 1995 23 5 1609 1629 10.1214/aos/1176324316

[B22] Self GS Liang KY Asymptotic Properties of Maximum Likelihood Estimators and Likelihood Ratio Tests under Nonstandard Conditions J Am Stat Assoc 1987 82 398 605 610 10.2307/2289471

