# Supplementary material for: Reanalysis of cancer mortality in Japanese A-bomb survivors exposed to low doses of radiation: bootstrap and simulation methods
Source: Environ Health. 2009 Dec 9;8:56. doi: 10.1186/1476-069X-8-56 (PMC2799447; doi:10.1186/1476-069X-8-56)
Supplement: Additional file 1 — Contains index file for downloading 4 Tables, 3 Code Files, 2 Commentary Files, and 5 Data Files. [file 1476-069X-8-56-S1.ZIP › index.html]

additional files


### Additional Files

**Files may be viewed by clicking on the links. However, it is preferable to save them as follows:**

*Make a new folder e.g. "twophase".*

right click on each of the links and use "Save Target as" to save the linked file to the "twophase" folder, without changing the filename.

### Tables (Excel)

S1  
S2  
S3  
S4

### Code Files (Text)

AC1.txt  
AC2.txt  
AC2s.txt

### Commentary Files (Word)

ACom1.doc  
ACom2.doc

### Data Files (Text)

stomdat1.txt  
lambda1.txt  
theta1.txt  
lambda2.txt  
theta2.txt

*If you would like to run the code file AC1.txt, first set **R** to open in the "twophase" folder (right click on the **R** icon and choose Properties, then adjust the Start in path). Open **R** and at the prompt, type:*

source("AC1.txt",echo=TRUE)

*if you then wish to run AC2.txt, quit and reopen **R** before typing the corresponding source line.*

On a 3 GHz PC AC1.txt takes about 1.5 mins and AC2.txt takes about 7 mins
