# Supplementary material for: Reanalysis of cancer mortality in Japanese A-bomb survivors exposed to low doses of radiation: bootstrap and simulation methods
Source: Environ Health. 2009 Dec 9;8:56. doi: 10.1186/1476-069X-8-56 (PMC2799447; doi:10.1186/1476-069X-8-56)
Supplement: Additional file 1 — Contains index file for downloading 4 Tables, 3 Code Files, 2 Commentary Files, and 5 Data Files. [file 1476-069X-8-56-S1.ZIP › ACom1.doc]

**ACom1: Commentary on AC1.txt**

The **R** code file AC1.txt is used to compute the bootstrap BC_a_ method 95% confidence intervals for ERR_1_ and ERR_0.025_ with the two-phase model for the stomach at the optimal latency, using 1000 replications. Lines beginning # are ignored by **R** but are pointers for this commentary.

Prior to running the BC_a_ code, the optimal latency is determined and the two-phase model is fitted to the low dose stomach data given in stomdat1.txt (the lengthy procedure for fitting the two-phase model is contained within the code in AC2.txt). This gives fitted values β = 0.45849085, σ = 119.95993465, τ = 82.66754681 along with the 17 fitted control parameters given in the file theta2.txt. The corresponding fitted Poisson parameters λ_i_T_i_ for each of the 3011 data cells are given in the file lambda2.txt

The code begins by reading these data files, setting beta1, sigma1, and tau1 as the fitted values of β, σ, and τ, setting the latency phi and defining the lagged dose dtse. The 3011 x 17 array cvar gives the covariates.

After *#check* the model is refitted holding β, σ, and τ at their MLE values as read; the resulting fitted control parameters should coincide with those in theta2.txt. During this stage the model is log-linear. Note that f, the quantity being minimised by the Newton-Raphson routine "optim", is a function of the 17-vector theta. It is obtained by summing lambda‑obs*log(lambda) over the data cells, where lambda is defined using theta. Up to a constant, f is the negative log likelihood of the data. The gradient of f is obtained as the matrix product of cvar with lambda-obs, since in each data cell ∂/∂β_j_(λ-obs(ln(λ))) = (1 - obs/λ)∂λ/∂β_j_ and for the control parameters ∂λ/∂β_j_ = λx_j_ where x_j_ are the covariates.

After *#hessian* the full two-phase model is refitted (briefly) to generate the hessian. As we already know the MLE, the lengthy procedure in AC2.txt where τ ranges over a succession of intervals, is replaced by a single step with τ > 0. The unknown 20-vector eta defines the control parameters along with beta and sigma, while tau is defined as exp(eta[20]). km is the function of eta to be minimised, and its gradient grm is obtained using cvar2p in place of cvar in the matrix product with lambda-obs. This reflects the formulas for ∂λ/∂β, ∂λ/∂σ, and ∂λ/∂log(τ). The hessian of the fitted model is denoted hess, and other fitted parameters and vectors are defined: betahat, sigmahat, and tauhat should coincide with the original values as read, while lambdahat is needed for the bootstrap.

After *#hesscheck* the computed hessian is briefly checked against the analytical form of the Fisher Information. The covariance matrix cov is obtained by inverting hess. The gradients of ERR_1_ and ERR_0.025_ are obtained as err1dot and err025dot, while dhat1 and dhat025 are their transformations by the covariance matrix.

The BC_a_ procedure (see Methods) begins after *#bootstrap.* For ERR_1_ the line "for (j in 1:B)" opens the main loop, which begins

obss<-rpois(3011,lambdahat)

This samples the 3011 Poisson distributions specified by lambdahat (using the fitted parameters) to give simulated observations obss. klf1 is a function of a single parameter, to be minimised along the line passing through the 20-parameter MLE for the original data with slope dhat1. Minimisation uses "optimise", which is preferable to "optim" for 1-parameter problems. The resulting values of beta, sigma, tau, and consequently ERR_1_ at the jth bootstrap are given by beta1boot[j] etc. The lines

score<-crossprod((obss-lambdahat),cvar2p)

scorelf[j]<-crossprod(t(score),dhat1)

yield the derivative of the log likelihood of the simulated data along the line with slope dhat1 through the MLE for the original data, computed at the MLE.

The main loop closes and after *#accbias*, the acceleration of the bootstrap sample is found from the 2^nd^ and 3^rd^ moments of scorelf, while the bias is computed by w<-qnorm(sum(err1boot<err1hat)/B).

The BC_a_ 95% confidence interval (see Methods) follows quickly, ending at bca1<-c(lo1,hi1).

After *#loop2* the entire process is repeated for ERR_0.025_ using a fresh bootstrap sample.

**Running AC1.txt**

If the additional files have been saved to the "twophase" folder (say), set **R** to open in this folder (right click on the **R** icon and choose Properties, then adjust the Start in path). Open **R** and at the prompt, type:

source("AC1.txt",echo=TRUE)

The hourglass should appear and the code will be visible when execution is completed. On a 3 GHz PC this takes about 1.5 mins.

nb: if AC2.txt has been run, quit and re-open **R** before running AC1.txt
