# Supplementary material for: Reanalysis of cancer mortality in Japanese A-bomb survivors exposed to low doses of radiation: bootstrap and simulation methods
Source: Environ Health. 2009 Dec 9;8:56. doi: 10.1186/1476-069X-8-56 (PMC2799447; doi:10.1186/1476-069X-8-56)
Supplement: Additional file 1 — Contains index file for downloading 4 Tables, 3 Code Files, 2 Commentary Files, and 5 Data Files. [file 1476-069X-8-56-S1.ZIP › ACom2.doc]

**ACom2: Commentary on AC2.txt**

The **R** code file AC2.txt is used to run the simulation in **R** for the stomach at (β_0_,σ_0_,τ_0_) = (0,0,1), at the optimal latency. In this example the value of τ_0_ is arbitrary. The code generates simulations of LRT_lin‑con_ and LRT_2p‑con_. The code here is set with n=3, to produce 3 lines of output. Lines beginning # are ignored by **R** but are pointers for this commentary.

At the outset, set.seed initialises the random number generator which is used in sampling to produce simulated data. The data file stomdat1.txt is read in; it was derived directly from the RERF original dataset LSS12, by restricting to the 0 – 20mSv subcohort. The files lambda1.txt and theta1.txt were generated separately by fitting the model to the original data, subject to the null hypothesis H_0_: (β,σ,τ) = (β_0_,σ_0_,τ_0_), i.e. in this case = (0,0,1). That fitting, not shown here, is straightforward as the model is log-linear in the remaining variables (see below re fitting the model to the simulated data subject to H_0_).

The code line “beta0=0” sets the null hypothesis, as the remainder of this code version presumes sigma0=0. cvar is an 3011x17 array whose rows give the values of the control covariates for each data cell. phi=11.89 sets the latency parameter at its optimal value (determined separately).

a=0 initialises the run to start with the first line of output. If the run is interrupted, it can be resumed by resetting a to the number of completed output lines and altering set.seed so the previous output is not duplicated. n=3 (here) gives the intended number of output lines.

After *#loop1*, the line “for (i in 1:n)” opens the main loop which starts

obs<-rpois(3011,lambda1)

This replaces the original observed data from stomdat1.txt by simulated data obtained from independent sampling of the Poisson distributions whose parameters are the 3011 values of the variable lambda1, defined from the file lambda1.txt. For each data cell, lambda1 is the expected number of stomach cancer deaths when the model is fitted subject to H_0_. Note that the simulated variable “obs” will vary as the main loop is re-run.

The next 29 lines fit the model to the simulated data subject to H_0_. f defines the function to be minimised by the subroutine “optim”, while gr is its gradient with respect to the remaining 17 parameters, having fixed β, σ and τ by the null hypothesis. Note that gr is evaluated by the line “2*crossprod((lambda-obs),cvar)” i.e. twice the matrix product of the array of control covariates with the column vector (lambda-obs). This formula arises because cvar is the gradient of lambda in this log-linear model.

At its minimum, f evaluates to the quantity K_0_ = -2Σ_1_^3011^(O~_i_lnλ~_0i_ - λ~_0i_) as defined in Methods. Optim begins searching from the parameter values theta1 obtained by fitting the model to the original data, subject to H_0_. Since f has a unique minimum, the choice of initial parameters is arbitrary.

After *#fit linear* the next 37 lines fit the model subject only to σ = σ_0_ = 0, i.e. β is no longer fixed at β_0_. Note that the definition of ERR has changed, as has the gradient. This section ends by computing LRT_lin-con_ as fitcon$value-fitlin$value.

Fitting the full model to the simulated data takes place in two stages. The first allows τ to vary freely (subject only to τ > 0) and uses an initial estimate obtained from fitting to the original data, subject to H_0_ . The second confines τ to 11 separate intervals followed by more exact minimisation within the preferred interval. The final outcome is the minimum of the two results.

After *#stage1* the next 80 lines form the first stage. ka is the function to be minimised by varying the 20 parameter vector etaa; taua is defined as exp(etaa[20]) > 0; and ERR = betaa*dtse+sigmaa*dtse*exp(-taua*dtse) is the full form of the model. The lines

if ((min(ERR)<(-0.999)))

{

etaa[18]=etaa[18]-4*(min(ERR)+0.999)

betaa<-etaa[18]

ERR<-betaa*dtse+sigmaa*dtse*exp(-taua*dtse)

}

which are repeated 3x, restore etaa to a permitted value if it strays over the boundary ERR > -1. gra, again a function of etaa, defines the gradient of ka including the repeated restoration if etaa strays over the boundary. gra is evaluated by “2*crossprod((lambda-obs),cvar2p)” with cvar2p, the gradient of lambda, defined in the previous line.

ka is now minimised by varying etaa, starting from the initial value “par=c(fitcon$par,beta0,0,0)” i.e. the values of the control parameters obtained by fitting the model to the simulated data subject to H_0_ , extended by beta=beta0 (=0 in this example), sigma=0, and tau=exp(0)=1.

Minimisation uses successive applications of various forms of “optim”: “Nelder-Mead” (the default), “Conjugate-Gradient”, and “BFGS”.

Almost all the remaining code is used to run stage 2, beginning at *#stage2*. This emulates the method in [2], restricting τ to the unit interval (0 , 1) and then to intervals of the form (2^m-2^, 2^m-1^) for 2 ≤ m ≤ 10, and finally to τ > 2^9^ . Preliminary optimisation is carried out in each interval and m is then reset to give the minimum of these 11 outputs. A final extended optimisation is carried out in a widened interval (0 , 2) if m=1 or (1.5*2^m‑3^, 1.5*2^m‑1^) otherwise. Much of the code at any value of m is similar to that in stage 1, however the array cvar2p, the gradient of lambda, is altered because tau is no longer defined as exp(eta[20]). For example during the preliminary optimisation when 2 ≤ m ≤ 10, tau = (2^m-2^)*(1+(u/(1+u))) where u = exp(eta[20]), and the last column of cvar2p is altered accordingly.

After *#compare stages*, the outputs of stage 1 and stage 2 are compared to choose the minimum, i.e. to maximise LRT_2p-con_ .

The main loop ends and, after *#write output*, the file out1.txt is generated.

If errors arise during the run the main loop will terminate but this is detected after writing the output and a call is then made to AC2s which replicates the code from *#start AC2s* to *#end AC2s*, initialised to begin where the loop ended. This call then leads to appending out1.txt and forms the last 22 lines of code, which can be repeated many times to cover anticipated errors (depending on the size of n).

In the current example with n=3, the output (shown to 4 decimal places) is:

| **ind** | **codec** | **codel** | **lrtlincon** | **betal** | **code2p** | **maxgr** | **lrt2pcon** | **errmin2p** | **betahat** | **sigmahat** | **tauhat** |
| --- | --- | --- | --- | --- | --- | --- | --- | --- | --- | --- | --- |
| 1 | 0 | 0 | 1.5061 | -0.0746 | 0 | 1.5238 | 2.4097 | -0.1300 | -0.0650 | 413.2330 | 419.6889 |
| 2 | 0 | 0 | 0.5430 | -0.0447 | 0 | 0.0141 | 1.2925 | -0.2410 | 0.1318 | -1.7449 | 2.1634 |
| 3 | 0 | 0 | 1.5124 | -0.0731 | 0 | 0.7193 | 11.1627 | 0 | 0.0857 | 124.4226 | 124.2642 |

The fields codec, codel, code2p, and maxgr are checks on the convergence, and errmin2p is the minimum value of ERR attained across all the data cells with the fitted two-phase model. betal is the fitted value of β in the linear model, likewise betahat etc. are the fitted parameters in the two-phase model. lrtlincon and lrt2pcon are LRT_lin-con_ and LRT_2p-con_ . LRT_2p-lin_ is obtained as LRT_2p-con_  - LRT_lin-con_ .

**Improvements**

In later computations, such as the simulations to determine variation in optimal latency, the approach to error handling and convergence was improved using tryCatch and parscale. For example the section of AC2

par[20]=log(v)

fitwop<-optim(par,kmx,control=list(maxit=1000,reltol=1e-8))

fitwop<-optim(fitwop$par,kmx,grmx,method="CG",control=list(maxit=50,reltol=1e-8))

fitwop<-optim(fitwop$par,kmx,grmx,method="BFGS",control=list(maxit=5,reltol=1e-8))

fitwop<-optim(fitwop$par,kmx,grmx,method="BFGS",control=list(maxit=10,reltol=1e-8))

fitwop<-optim(fitwop$par,kmx,grmx,method="BFGS",control=list(maxit=20,reltol=1e-8))

fitwop<-optim(fitwop$par,kmx,grmx,method="BFGS",control=list(maxit=2000,reltol=1e-8))

fitwop<-optim(par,kmx,control=list(maxit=10000,reltol=1e-8))

fitwop<-optim(fitwop$par,kmx,grmx,method="BFGS",control=list(maxit=2000,reltol=1e-8))

may be replaced by

par[20]=log(v)

pars<-abs(par)+1

fitt<-tryCatch({fitwop<-optim(par,kmx,control=list(maxit=1000,reltol=1e-8,parscale=pars))

fitwop<-optim(fitwop$par,kmx,grmx,method="CG",control=list(maxit=50,reltol=1e-8))

fitwop<-optim(fitwop$par,kmx,grmx,method="BFGS",control=list(maxit=5,reltol=1e-8))

fitwop<-optim(fitwop$par,kmx,grmx,method="BFGS",control=list(maxit=10,reltol=1e-8))

fitwop<-optim(fitwop$par,kmx,grmx,method="BFGS",control=list(maxit=20,reltol=1e-8))

fitwop<-optim(fitwop$par,kmx,control=list(maxit=10000,reltol=1e-8,parscale=pars))

if (fitwop$convergence!=0)

{fitwop<-optim(fitwop$par,kmx,control=list(maxit=10000,reltol=1e-8,parscale=pars))}

fitwop}, error=function(ex)

{fitwopc<-optim(par,kmx,control=list(maxit=1000,reltol=1e-8))

fitwopc<-optim(fitwopc$par,kmx,grmx,method="CG",control=list(maxit=50,reltol=1e-8))

fitwopc<-optim(fitwopc$par,kmx,grmx,method="BFGS",control=list(maxit=5,reltol=1e-8))

fitwopc<-optim(fitwopc$par,kmx,grmx,method="BFGS",control=list(maxit=10,reltol=1e-8))

fitwopc<-optim(fitwopc$par,kmx,grmx,method="BFGS",control=list(maxit=20,reltol=1e-8))

fitwopc<-optim(fitwopc$par,kmx,control=list(maxit=10000,reltol=1e-8))

if (fitwopc$convergence!=0)

{fitwopc<-optim(fitwopc$par,kmx,control=list(maxit=10000,reltol=1e-8))}

fitwopc})

This rescales the parameters using parscale, improving convergence of optim for the default Nelder-Mead method, but if an error is generated the alternative code without parscale is called.

**Running AC2.txt**

If the additional files have been saved to the "twophase" folder (say), set **R** to open in this folder (right click on the **R** icon and choose Properties, then adjust the Start in path). Open **R** and at the prompt, type:

source("AC2.txt",echo=TRUE)

The hourglass should appear and the code will be visible when execution is completed. On a 3 GHz PC this takes about 7 mins (for 3 lines of output)

nb: if AC1.txt has been run, quit and re-open **R** before running AC2.txt
